# Similar Genetic Basis of Resistance to Bt Toxin Cry1Ac in Boll-Selected and Diet-Selected Strains of Pink Bollworm

**DOI:** 10.1371/journal.pone.0035658

**Published:** 2012-04-18

**Authors:** Jeffrey A. Fabrick, Bruce E. Tabashnik

**Affiliations:** 1 USDA-ARS, U.S. Arid Land Agricultural Research Center, Maricopa, Arizona, United States of America; 2 Department of Entomology, University of Arizona, Tucson, Arizona, United States of America; Cinvestav, Mexico

## Abstract

Genetically engineered cotton and corn plants producing insecticidal *Bacillus thuringiensis* (Bt) toxins kill some key insect pests. Yet, evolution of resistance by pests threatens long-term insect control by these transgenic Bt crops. We compared the genetic basis of resistance to Bt toxin Cry1Ac in two independently derived, laboratory-selected strains of a major cotton pest, the pink bollworm (*Pectinophora gossypiella* [Saunders]). The Arizona pooled resistant strain (AZP-R) was started with pink bollworm from 10 field populations and selected with Cry1Ac in diet. The Bt4R resistant strain was started with a long-term susceptible laboratory strain and selected first with Bt cotton bolls and later with Cry1Ac in diet. Previous work showed that AZP-R had three recessive mutations (*r1*, *r2*, and *r3*) in the pink bollworm cadherin gene (*PgCad1*) linked with resistance to Cry1Ac and Bt cotton producing Cry1Ac. Here we report that inheritance of resistance to a diagnostic concentration of Cry1Ac was recessive in Bt4R. In interstrain complementation tests for allelism, F_1_ progeny from crosses between AZP-R and Bt4R were resistant to Cry1Ac, indicating a shared resistance locus in the two strains. Molecular analysis of the Bt4R cadherin gene identified a novel 15-bp deletion (*r4*) predicted to cause the loss of five amino acids upstream of the Cry1Ac-binding region of the cadherin protein. Four recessive mutations in *PgCad1* are now implicated in resistance in five different strains, showing that mutations in cadherin are the primary mechanism of resistance to Cry1Ac in laboratory-selected strains of pink bollworm from Arizona.

## Introduction

Insecticidal crystalline proteins from the common soil bacterium *Bacillus thuringiensis* (Bt) kill some insect pests, but cause little or no harm to most non-target organisms including people [1]–[3]. Genetically engineered crops producing Bt proteins for insect control were first cultivated commercially in 1996 [3] and grew on more than 58 million hectares worldwide in 2010 [4]. Such Bt crops can improve yields and reduce reliance on conventional insecticides, thereby providing economic, health, and environmental benefits [5]–[8]. However, the evolution of resistance to Bt crops by insect pests can reduce such benefits.

Field-evolved resistance to Bt crops has been reported for some populations of several insect pests [9]–[15]. Although the mechanisms of resistance have not been reported for these cases, they have been identified in many laboratory-selected strains and in pest populations that evolved resistance outside of the laboratory to the Bt toxins used in sprays [2], [16]–[19]. The most common mechanism involves changes in larval midgut target sites that reduce binding of Bt toxins [20]–[25].

Here we focus on resistance to Bt toxin Cry1Ac in the pink bollworm, *Pectinophora gossypiella* (Saunders), one of the world's most destructive pests of cotton worldwide [26]. In western India, pink bollworm resistance to Bt cotton producing Cry1Ac is associated with widespread control failures of this crop [13]–[14]. In China, field control failures have not been reported, but pink bollworm susceptibility to Cry1Ac has decreased significantly [27]. By contrast, field populations of pink bollworm have remained susceptible to Cry1Ac in Arizona for 15 years, enabling use of Bt cotton producing either Cry1Ac alone or Cry1Ac and Cry2Ab as a primary tool of a multi-tactic eradication program [28]–[29].

Although Arizona field populations remain susceptible, several laboratory strains of pink bollworm from Arizona were selected for resistance to Cry1Ac by rearing them on diet treated with this toxin [30]–[32]. In all of these strains, including the Arizona pooled resistant strain (AZP-R), resistance to Cry1Ac and to Bt cotton producing Cry1Ac is linked with mutations in the *PgCad1* gene (previously called *BtR*) that encodes a Cry1Ac-binding cadherin protein [21], [33]–[35]. Moreover, in all of these diet-selected strains, resistance is associated with up to three recessive resistance alleles (*r1*, *r2*, and *r3*) of *PgCad1* that carry mutations expected to disrupt the cadherin protein [21], [32]–[33].

Here we determined the genetic basis of resistance to Cry1Ac in the Bt4R strain of pink bollworm, which was first selected for resistance on Cry1Ac-producing Bt cotton bolls for 42 generations, followed by selection for more than 15 generations on diet treated with Cry1Ac [36]–[37]. Previous results showed that the Bt4R strain lacked the *r1*, *r2*, and *r3* alleles found in resistant strains that had been selected only on diet treated with Cry1Ac [37]. This led to the hypothesis that the initial selection on bolls might have yielded a different mechanism of resistance than selection exclusively on Cry1Ac-treated diet [37]. The results reported here from complementation tests for allelism show that the same locus confers resistance in Bt4R and AZP-R. Molecular analysis revealed a new mutant cadherin allele (*r4*) in Bt4R that has a deletion expected to disrupt the cadherin protein.

## Results

### Dominance and Complementation Test for Allelism

**Figure 1:**
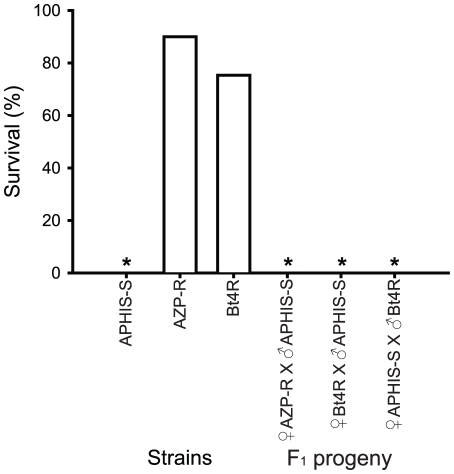
Survival at the diagnostic concentration of Cry1Ac of pink bollworm larvae from two resistant strains (AZP-R and Bt4R), a susceptible strain (APHIS-S), and their F_1_ progeny from mass crosses. Asterisks indicate 0% survival for the APHIS-S strain and the F_1_ progeny of the three crosses with APHIS-S.

Survival at the diagnostic concentration of Cry1Ac (10 µg Cry1Ac per mL diet) was 90% for resistant strain AZP-R and 0% for the susceptible strain APHIS-S (Fig. 1), confirming previous results [21], [33]. Survival of resistant strain Bt4R was 75% (Fig. 1), which suggests that it might have included a mixture of resistant and susceptible individuals.

Survival of F_1_ progeny from mass crosses between Bt4R and a susceptible strain did not differ between reciprocal crosses (i.e., ♀ Bt4R×♂ APHIS-S versus ♀ APHIS-S×♂ Bt4R), indicating autosomal inheritance (Fig. 1). Survival was 0% for the F_1_ progeny from crosses between Bt4R and a susceptible strain, indicating completely recessive resistance (*h* = 0) at the diagnostic concentration (Fig. 1). Survival was also 0% for the F_1_ progeny of a cross between AZP-R and the susceptible strain (Fig. 1), which confirmed that resistance of AZP-R was completely recessive at the diagnostic concentration [33], [38]–[39].

**Figure 2:**
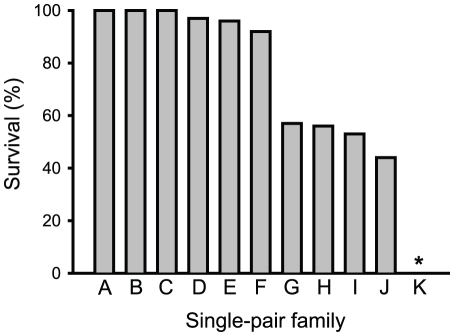
Survival at the diagnostic concentration of Cry1Ac of F_1_ progeny from single-pair crosses between resistant strains Bt4R and AZP-R. Families A, E, F, G, I, and J from female AZP-R×male Bt4R and B, C, D, and K from female Bt4R×male AZP-R. The asterisk indicates 0% survival for family K.

In the F_1_ progeny of 11 families generated from single-pair crosses between Bt4R and AZP-R, survival was close to 100% for six families (A–F, mean = 98%, range = 92 to 100%), close to 50% for four families (G–J, mean = 52%, range = 44 to 57%), and 0% for one family (K) (Fig. 2). Together with data showing that resistance was completely recessive in Bt4R and AZP-R (Fig. 1), the results from the six families with close to 100% survival (Fig. 2) imply that recessive alleles at the same locus confer resistance in AZP-R and in these six families from Bt4R.

A potential explanation for the ∼50% survival in families G–J and 0% survival in family K, is that Bt4R, AZP-R, or both contained a mixture of resistant (*rr*), heterozygous (*rs*) and susceptible individuals (*ss*). The survival of 90% for AZP-R and 75% for Bt4R suggests that heterogeneity was greater in Bt4R (Fig. 1). In addition, Bt4R had not been selected with Cry1Ac during the 24 generations immediately preceding the single-pair cross experiment. Thus, we hypothesized that the AZP-R parents were homozygous for resistance at the cadherin locus (*rr*), while the Bt4R parents were *rr* in the six families with close to 100% survival, *rs* in the families with close to 50% survival (expected progeny: 50% *rr* and 50% *rs*), and *ss* in the family with 0% survival. To test this hypothesis, we checked the cadherin allele from Bt4R for mutations, developed a PCR method for screening for the mutation in Bt4R, and screened parents and offspring from the single-pair crosses for this mutation, as described below.

### Cadherin Allele *r4* from Bt4R

Compared with *PgCad1* from the susceptible APHIS-S strain ([21]; AY198374) that encodes a predicted cadherin protein with 1,735 amino acids, cadherin cDNA isolated from the resistant Bt4R strain had a 15-bp deletion resulting in a predicted protein with five fewer amino acids (943-IDLDS-947) (Fig. S1; JQ279500.1). We named this mutation and the allele in which it occurs *r4*.

Aside from the five missing amino acids in Bt4R, the cadherin proteins in susceptible pink bollworm and Bt4R are expected to have generally similar structures, including a secretion signal peptide, 11 extracellular cadherin repeats, a membrane proximal domain, and an intracellular domain [21], [34]. However, 22 nucleotide substitutions exist of which 14 result in changed amino acids (Fig. S2). These 14 substitutions are S18L, L212P, F299S, F341L, V440A, I469T, D599G, Q613R, N623D, I647T, Q949E, S981R, N1188S, and D1511G (Fig. S3). These substitutions are mainly conservative changes, and have not been shown to be associated with resistance in other *PgCad1 r* alleles. Interestingly, two of the four cDNA sequences had an additional missing 3-bp (nucleotides 122–124), resulting in the loss of Ser26 and the S27L substitution (data not shown). However, this change was not found in all *PgCad1* clones from different resistant individuals and thus is not linked with resistance.

### PCR Detection of Cadherin Allele *r4*

**Figure 3:**
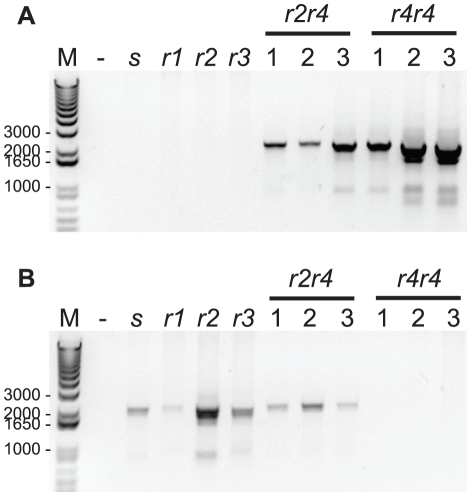
PCR-based detection of the *r4* cadherin resistance allele. Genomic DNA extracted from pink bollworm individuals with different genotypes was subjected to *r4* and *r4x* PCR genotyping. (A) The *r4* allele was PCR amplified using 156PgCad5 and 158PgCad3 primers from all individuals with *r4* allele, but not from individuals with *ss*, *r1r1*, *r2r2*, or *r3r3* cadherin genotypes. (B) The *r4x* reaction allows for discrimination between *r4r4* resistant homozygotes and heterozygotes with a single *r4* allele. PCR primers 159PgCad5 and 158PgCad3 correspond to missing region of the *r4* mutation and will amplify all alleles except *r4*. The genotypes *ss*, *r1r1*, *r2r2*, and *r3r3* are indicated as *s*, *r1*, *r2*, and *r3*. DNA from a total of three *r2r4* and three *r4r4* individuals (1, 2, and 3) are shown. No template control PCR reaction is shown in “-” lane. Lane M contains 1 Kb Plus DNA Ladder (Invitrogen) and corresponding sizes are shown.

We developed an allele-specific PCR reaction using primers 156PgCad5 and 158PgCad3 to amplify gDNA corresponding to the *r4* cadherin mutation (i.e., *r4* reaction). Whereas 156PgCad5 spans the 15-bp deletion and is specific for the *r4* mutation, 158PgCad3 anneals to a sequence that is conserved in all known *PgCad1* alleles (see Figures S1 and S2). As expected, an ∼2 kb PCR product was amplified from gDNA obtained from individuals containing the *r4* allele, but not from gDNA from individuals with genotypes *ss*, *r1r1*, *r2r2*, or *r3r3* (Fig. 3A).

To discriminate between genotypes *r4r4* and *r4x* (where *x* represents any allele other than *r4*), we used primers 159PgCad5 and 158PgCad3 (i.e., *r4x* reaction). 159PgCad5 corresponds to the altered nucleotide sequence of the *r4* mutation, including the 15-bp deletion and the adjacent 2-bp substitution (Figure S2). Because the majority of the primer sequence is missing from *r4* genomic DNA, a positive *r4x* reaction indicates the presence of an allele other than *r4*. While *r4r4* individuals yielded a band of ∼2 kb in the *r4* reaction and no band in the *r4x* reaction, *r2r4* individuals yielded a band of ∼2 kb in both the *r4* reaction and the *r4x* reaction (Fig. 3B). PCR analysis of the progeny from mass crosses that survived exposure to the diagnostic concentration of Cry1Ac showed that all survivors genotyped from Bt4R (n = 28) were *r4r4* and all survivors genotyped from AZP-R (n = 31) were *r2r2*.

### Genotypes of Parents and Offspring from Single-Pair Crosses

As expected, all parents from AZP-R in the 11 single-pair crosses were *r2r2*. We were able to genotype 9 of the 11 parents from Bt4R. All nine of these Bt4R parents had the expected genotype: five *r4r4* parents yielded the five families with survival close to 100%, three *r4s* parents yielded the three families with close to 50% survival, and one *ss* parent yielded the family with 0% survival. Also, as expected, all progeny genotyped from single-pair crosses between Bt4R and AZP-R that survived exposure to the diagnostic concentration were *r2r4* (n = 147).

### Survival of Bt4R Larvae on Bt Cotton Plants

**Figure 4:**
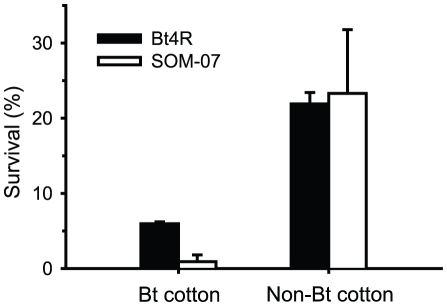
Survival (mean and SE) in greenhouse bioassays with resistant (Bt4R) and susceptible (SOM-07) strains of pink bollworm on Bt cotton and non-Bt cotton plants.

In greenhouse bioassays (Fig. 4), survival on Bt cotton plants was significantly higher for Bt4R (mean = 6.0%, SE = 0.2%) than for the unselected strain SOM-07 (mean = 0.9%, SE = 0.9%) (t-test, t = 5.4, df = 2, P = 0.03). On non-Bt cotton plants, survival was similar between Bt4R (mean = 21.8%, SE = 1.6%) and SOM-07 (mean = 23.2%, SE = 8.4%) (t-test, t = 0.2, df = 2, P = 0.9). Mean survival on Bt cotton relative to non-Bt cotton was 0.28 for Bt4R compared with 0.04 for SOM-07. In the first trial of the greenhouse bioassay, all seven bolls from five Bt cotton plants in which nine Bt4R larvae survived tested positive for Cry1Ac based on a commercial Cry1Ac strip test. In the second trial, all ELISA tests of seeds from 10 Bt cotton bolls in which 15 Bt4R larvae survived were positive for Cry1Ac (mean = 0.21 µg Cry1Ac per g dried seed material, SE = 0.01). In the second trial, all five seeds tested from five different non-Bt cotton plants lacked Cry1Ac (mean = −0.004 µg Cry1Ac per g dried seed material, SE = 0.001).

## Discussion

The results here show that recessive mutations in the cadherin gene are associated with resistance to Bt toxin Cry1Ac in two strains of pink bollworm, AZP-R and Bt4R, that differ in their origin and the way in which they were selected for resistance in the laboratory. While AZP-R was started with individuals collected in 1997 from 10 Arizona field populations and selected exclusively on diet with Cry1Ac [38]–[39], Bt4R was started with a long-term susceptible laboratory strain and selected first for 42 generations by feeding larvae for four days on Bt cotton bolls and later with Cry1Ac in diet [36]–[37]. Survival of Bt4R at the diagnostic concentration of Cry1Ac was 58% after selection only on bolls for 35 generations [36] and increased to 75% after 15 generations of selection with Cry1Ac in diet (Fig. 1).

The results here show completely recessive resistance of both strains to a diagnostic concentration of Cry1Ac, which confirms previous results with AZP-R [33], [38]–[40], but differs from previous results with Bt4R indicating partially recessive resistance [37]. Here survival of F_1_ progeny from both reciprocal mass crosses between Bt4R and the susceptible APHIS-S strain was 0%, whereas survival was 1% in the F_1_ progeny from a mass cross between Bt4R females and males from the susceptible WCRL strain, and 9% in the F_1_ progeny from a mass cross between WCRL males and Bt4R females [37]. Several factors could have produced the low, but >0% survival in the previous tests including a low proportion of individuals in WCRL that were heterozygous for resistance or inclusion of incorrectly sexed Bt4R individuals in the mass crosses, leading to one or more Bt4R×Bt4R matings instead of the intended Bt4R×WCRL matings. We also cannot rule out the possibility of a rare non-recessive allele in the previous tests that was lost from Bt4R before the experiments reported here were conducted.

The results here show that, unlike previous results with Bt4R [37], larvae from Bt4R survived on Bt cotton plants producing Cry1Ac. After selection for only 4 days on Cry1Ac-producing Bt cotton bolls in each of 42 generations from 2001–2005, followed by selection during 11 generations on diet containing 10 µg Cry1Ac per mL diet from 2006–2007, Bt4R larvae could not complete their development on Cry1Ac-producing Bt cotton bolls [37], which requires >21 days [30], [40]. Additional selection during 2007–2010 on diet containing 10 to 300 µg Cry1Ac per mL diet could have increased resistance of Bt4R to Cry1Ac and survival on Bt cotton bolls producing Cry1Ac. Tests for survival of Bt4R on Bt cotton were performed previously on the NuCOTN33B® variety [36]–[37] and here on DP 449 BG®/RR®, a Bt cotton variety grown commercially in the US beginning in 2004. Thus, survival on Bt cotton in this study, but not in previous tests, could also reflect a difference between these varieties.

Survival on non-Bt cotton cultivar DP5415 was not significantly lower for Bt4R than for the unselected strain SOM-07, which indicates we detected no fitness cost reducing larval survival of Bt4R on non-Bt cotton. This confirms previous results with DP5415, which show survival on this cultivar was not significantly lower for larvae with resistance alleles than for larvae without resistance alleles [32], [41]. By contrast, in most experiments with non-Bt cotton cultivar DP50, survival was significantly lower for resistant than susceptible larvae [40], [42]–[44], [ but see 38]. Collectively, these results support the hypothesis that the cultivar of non-Bt cotton affects fitness costs [41].

One might predict that fitness costs would be higher for the *r2* cadherin resistance allele of pink bollworm, which has a premature stop codon, than for the *r1*, *r3* and *r4* cadherin resistance alleles, which do not have a premature stop codon, but instead have deletions of 24, 126 and 15 bp, respectively [21]. Previous results, however, are not consistent with this hypothesis [41]. On non-Bt cotton plants, larval survival was not lower for *r2r2* than for *r1r3* or *r3r3*, and pupal weight for *r2r2* was intermediate between *r1r1* and *r3r3* [41]. In addition, fitness costs for the *r3* allele with a 126-bp deletion were not consistently higher than for the *r1* allele with a 24-bp deletion [41], [45].

As with diet-selected resistant strains of pink bollworm [41], Bt4R had lower survival on Bt cotton than non-Bt cotton, which indicates incomplete resistance. Survival on Bt cotton relative to non-Bt was only 0.28 for Bt4R, compared with a mean of 0.79 (range: 0.40 to 1.5) for several previously tested diet-selected strains of pink bollworm [41]. Because Bt4R had a mixture of susceptible and resistant individuals, as indicated by bioassays and DNA sequencing, the comparisons here may underestimate the fitness cost associated with *r4* and the survival of *r4r4* larvae on Bt cotton relative to non-Bt cotton. Based on the bioassay data showing survival of 75% of Bt4R larvae at the diagnostic concentration of Cry1Ac, we estimate that 75% of the Bt4R larvae tested on Bt cotton were *r4r4* homozygotes, which yields an adjusted survival on Bt cotton relative to non-Bt cotton of 0.37 for *r4r4* homozygotes (0.28/0.75).

Here we identified a new mutation (*r4*) in the *PgCad1* cadherin gene from the Bt4R strain that is a 15-bp deletion, predicted to eliminate five amino acids in the seventh cadherin repeat (CR7) of the wild-type cadherin protein. This region does not bind Cry1Ac [34], which implies that the *r4* mutation does not affect binding directly. Like *r4*, the *r1* and *r3* pink bollworm cadherin resistance alleles affect a region that is not involved directly in toxin binding (CR9) [21], [35]. In addition, relative to a susceptible strain, binding to brush border membrane vesicles from AZP-R was reduced for Cry1Ab, but not Cry1Ac [46]. Thus, deletion of some amino acids upstream of the putative binding region could modify post-binding events without preventing toxin binding [46]. Functional studies of the cadherin proteins encoded by wild type and mutant alleles in conjunction with analysis of binding and post-binding events in susceptible and resistant larvae are needed to test this hypothesis. The *r1*, *r3* and *r4* cadherin resistance alleles of pink bollworm differ from several cadherin resistance alleles that have premature stop codons upstream of toxin-binding regions: the *r2* mutation in pink bollworm [21], the *r1* mutation in *Heliothis virescens* cadherin (*HevCaLP*) [20], and the *r1*-*r8* mutations in *Helicoverpa armigera* [22], [47]. The truncated cadherins encoded by alleles with these mutations are not expected to bind Cry1Ac.

Although each of the two pink bollworm strains examined in this study had a unique history, both originated in Arizona. Whereas the AZP-R strain was founded from pink bollworm collected at 10 sites throughout Arizona in 1997 [31], the Bt4R strain was derived from a susceptible strain (WCRL) that had been reared in the laboratory for several decades without exposure to Cry1Ac. For AZP-R, two rounds of selection on Cry1Ac greatly increased resistance to Cry1Ac [38], while Bt4R was selected for many generations on Bt cotton bolls and on Cry1Ac in diet before it became highly resistant [36]–[37]. Like Bt4R, the diet-selected resistant strain APHIS-98R was derived from a long-term susceptible laboratory strain (APHIS-S) [48]. However, in contrast to Bt4R with its *r4* cadherin allele, resistance to Cry1Ac and Bt cotton in APHIS-98R was linked with *r1*, *r2*, and *r3* cadherin alleles [33]. The different resistance alleles found in Bt4R and APHIS-98R reflect their origins from different parental strains; APHIS-98R was derived from APHIS-S [48] and Bt4R was derived from WCRL [36]. In two other resistant strains of pink bollworm examined previously, the *r1* and *r3* alleles occurred in the MOV97-R strain from Mohave Valley in western Arizona, and the *r1* and *r2* alleles in the SAF97-R strain from Safford in eastern Arizona [21]. Counting AZP-R, APHIS-98R, MOV97-R, SAF97-R, and Bt4R, resistance alleles at the cadherin locus *PgCad1* have been detected in five distinct laboratory-selected strains of pink bollworm. These results show that cadherin resistance alleles provide the primary basis of resistance to Cry1Ac in laboratory-selected strains of pink bollworm from Arizona. It remains to be determined if cadherin resistance alleles are important in field-evolved resistance to Cry1Ac in India or China or elsewhere.

## Materials and Methods

### Pink Bollworm Strains

We used four strains of pink bollworm from Arizona: APHIS-S, SOM-07, AZP-R, and Bt4R. APHIS-S is a susceptible strain that had been reared in the laboratory for more than 30 years without exposure to Bt toxins [40], [49]. SOM-07 is an unselected strain that was started with pink bollworm collected in 2007 from Somerton, Arizona and reared in the lab without exposure to Bt toxins. When tested in 2007, SOM-07 had 0% survival at the diagnostic concentration (10 µg Cry1Ac per mL diet) (n = 340).

AZP-R and Bt4R are laboratory-selected resistant strains. AZP-R was started by pooling survivors of exposure to various concentrations of Cry1Ac in diet from 10 strains derived in 1997 from 10 Arizona cotton fields [38]. Additional selection with Cry1Ac in wheat germ diet was done with AZP-R [38]–[39]. The concentration of Cry1Ac killing 50% of larvae (LC_50_, in µg Cry1Ac per mL diet) was previously reported for AZP-R as 700 [39] to 2100 [50], which was 1500 to 3100-fold higher than for the concurrently tested susceptible APHIS-S strain [39], [50]. Whereas AZP-R initially had all three cadherin alleles (*r1*, *r2*, and *r3*) with *r2* as the most common allele [21], [33], more recent results reported previously [35] and here show that only the *r2* allele was detected in AZP-R.

Bt4R originated from the WCRL strain that had been reared on artificial diet at the Western Cotton Research Laboratory, Phoenix, AZ for >300 generations without exposure to Cry1Ac or Bt cotton [36]. A substrain of WCRL called BG(4) strain was selected for resistance by feeding larvae that were 4 to 5 days old on bolls of Bt cotton producing Cry1Ac for four days, then transferring them to untreated artificial diet in 42 generations [36]–[37]. Bt4R is a substrain of BG(4) that was selected for >11 generations on artificial diet containing 10 µg of Cry1Ac toxin per milliliter diet, pooling survivors from a selection on either 32 or 100 µg Cry1Ac per mL diet, and subsequent selection of a single generation on 10, 100, and 300 µg of Cry1Ac per milliliter diet. Before Bt4R was selected on diet containing >10 µg of Cry1Ac per mL diet, the LC_50_ for Bt4R was 61 µg Cry1Ac per mL diet, which was 240-fold higher than the LC_50_ for the susceptible WCRL strain [37].

During the 24 generations (about two years) immediately before the complementation test, Bt4R was reared on diet without Cry1Ac. Aside from the four-day feeding on cotton bolls mentioned above, all larvae were reared on wheat germ diet [51] at 27 °C with 14 h light∶10 h dark.

### Diet Bioassays

Newly hatched neonates were put individually in 30 mL plastic cups with 3 g diet with either 0 (untreated) or 10 µg Cry1Ac per mL diet [38], [40]. The source of Cry1Ac was MVPII (Dow Agrosciences, San Diego, CA), a liquid formulation containing protoxin encapsulated in *Pseudomonas fluorescens* [39]. After 21 d, live fourth instar larvae, pupae, and adults were scored as survivors.

### Mass Crosses: Maternal Effects, Sex Linkage, and Dominance

We conducted two types of mass crosses between strains: AZP-R×APHIS-S and Bt4R×APHIS-S. For Bt4R×APHIS-S, we conducted each of the two reciprocal crosses (♀ Bt4R×♂ APHIS-S and ♀ APHIS-S×♂ Bt4R). For AZP-R×APHIS-S, which has been examined extensively before [33], [38]–[39] and was included here as an internal control, we did only one of the two reciprocal crosses (♀ AZP-R×♂ APHIS-S). The sex of each pupa was determined visually. For each mass cross (e.g., ♀ Bt4R×♂ APHIS-S, etc), 35 female pupae from one strain were pooled with 35 male pupae in 30 mL cardboard containers and held at 27 °C with 14∶10 h light∶dark cycle. Newly eclosed adults were provided 10% sucrose and a 2 cm^2^ piece of Whatmann filter paper placed over meshed-screen provided an oviposition substrate. Sample sizes for bioassays testing the F_1_ progeny from mass crosses were 50 on treated diet and 20 on control diet.

To evaluate maternal effects and sex linkage of resistance in Bt4R, we compared survival of F_1_ progeny between the two reciprocal crosses between Bt4R and APHIS-S [48]. To evaluate dominance, we compared survival of Bt4R, APHIS-S, and their F_1_ progeny [52].

### Single-Pair Crosses: Interstrain Complementation Test for Allelism

To determine if the locus or loci conferring resistance to Cry1Ac differed between AZP-R and Bt4R, we performed interstrain complementation tests for allelism [53]. If two resistant strains are crossed, each with recessive alleles for resistance at unique loci, susceptibility (the wild-type phenotype) will be restored in the progeny due to allelic complementation. However, if the recessive resistance alleles occur at the same locus, progeny will be resistant because they will inherit resistance alleles at the same locus from both parents. We used the bioassay method described above to test the F_1_ offspring from crosses between the two resistant strains (AZP-R×Bt4R) and between APHIS-S and Bt4R.

We conducted single-pair reciprocal crosses between the AZP-R and Bt4R strains using methods similar to those for the mass crosses, except that each pair was set up with one male pupa from a resistant strain and one female pupa from the other resistant strain in a 30 mL cup. Sample sizes for bioassays testing the F_1_ progeny from 11 single-pair crosses were 14 to 30 neonates per family on treated diet and 7 to 20 larvae per family on untreated diet. Sample sizes for bioassays of F_1_ progeny from 10 single-pair reciprocal crosses for APHIS-S×Bt4R were 26 to 30 neonates per family on treated diet and 17 to 20 neonates per family on untreated diet.

Tests on diet containing a diagnostic concentration of Cry1Ac revealed that Bt4R consists of a mixture of resistant and susceptible individuals, which was advantageous because the inheritance of resistance was clearly defined. Had the parents of single-pair crosses been homozygous resistant (i.e. *rr*), all F_1_ progeny would have been expected to survive at the diagnostic Cry1Ac concentration. However, because no survival was observed in F_1_ offspring from *ss* or *rs* parents, we can be certain that no non-recessive resistance genes were missed.

### *PgCad1* cDNA Cloning

For cDNA cloning of *PgCad1*, we used five 4th instars from the Bt4R strain that survived exposure to the diagnostic concentration of Cry1Ac and thus were expected to be homozygous resistant. We extracted total RNA using TRIzol® reagent (Invitrogen, Carlsbad, CA) according to manufacturer's instructions. RNA concentration was determined using NanoDrop ND1000 spectrophotometer (Thermo Scientific, Wilmington, DE) and total RNA quality was accessed on the Agilent BioAnalyzer 2100 with RNA Nano 6000 LabChip Kit (Agilent Technologies, Santa Clara, CA). cDNA was produced using random hexamer primers and ThermoScript RT-PCR System (Invitrogen) according to manufacturer's recommendations. Full-length *PgCad1* was amplified from two individuals in PCR using SuperTaq Plus DNA Polymerase (Ambion, Austin, TX) and primers 52PgCad5 (5-ATGGCGGGTGACGCCTGCATAC-3′) and 25PgCad3 (5′-CTATGGTCGCATGCGCCTGTTAGT-3′), which correspond to the 5′- and 3′-ends of *PgCad1* (AY198374.1). PCR products were A-tailed with 1 U of Takara Ex Taq (Takara Bio USA, Madison, WI) and separated on an 0.8% agarose gel stained with Crystal Violet (Invitrogen). Bands were gel-purified and inserted into pCR-XL-TOPO using TOPO XL Gel Purification and PCR cloning kits (Invitrogen). Plasmids were propagated in OneShot TOP10 electrocompetent *Escherichia coli* (Invitrogen) and purified using QIAprep Spin MiniPrep kit in QIAcube robot (Qiagen, Valencia, CA). Inserts were sequenced at the using T7 vector primer (5′-TAATACGACTCACTATAGGG-3′), 52PgCad5, 70PgCad5 (5′-GACCGCCGCGATGGATGGAAAT-3′), 72PgCad5 (5′-GACTGTACCCAAGGACTATCACGTCGG-3′), 76PgCad5 (5′-CTGAACCAGACCTTCAGTATTCGGGAG-3′), 78PgCad5 (5′-AAATGCACCCGATTTCACAAACGTG-3′), 79PgCad3 (5′-TTAGGCGACAGCATGTTGAGAAGTCTC-3′), 81PgCad3 (5′-GGAGCGAGAACCTCTCAGTCAAGCC-3′), 85PgCad3 (5′-GAAGGACACCCTATTTTGGGAT-3′), 25PgCad3, and M13 reverse vector primer (5′-CAGGAAACAGCTATGAC-3′). The nucleotide sequence reported in this paper is deposited in the GenBank public database with accession number JQ279500.1.

### PCR Genotyping

Genomic DNA was extracted from individual larvae, pupae, and adults using the PUREGENE DNA Isolation Kit (Qiagen, Valencia, CA) and DNA screening for the presence of known cadherin resistance alleles (*r1*, *r2*, and *r3*) was done using a modified protocol and PCR primers of that described by Morin et al. [54] and Tabashnik et al. [32]. Survivors on Cry1Ac-treated diet from Bt4R×Bt4R (n = 32) and AZP-R×AZP-R (n = 45) mass crosses were PCR genotyped. Parents of the eleven informative families used in the single pair reciprocal crosses (n = 22 adults) as well as the survivors from each family on 10 µg Cry1Ac per mL diet were genotyped. To check the quality of gDNA at the cadherin locus, we measured the amplification of the ∼1,600 bp “X band” from the *r3x* reaction [54]. Furthermore, DNA previously extracted from diet-selected resistant strains containing known *r* alleles (*r1r3* and *r2r3*) were used as positive controls for genotyping (as described in Morin et al. [21], [54]).

Following the PCR logic for detection of *r1*, *r2*, and *r3* cadherin resistance alleles [32], [54], we developed a PCR assay for detection of the *r4* cadherin allele. PCR of the r4 mutation was performed using Takara Ex Taq DNA polymerase and primers 156PgCad5 (5′-CGAGACTTCTTCGCCGGTGA-3′) and 158PgCad3 (5′-TCTGGAGTCGGCAATTCAGGAG-3′). Whereas the sequence corresponding to 158PgCad3 does not differ between the susceptible allele (*s*) or the *r* alleles (*r1*, *r2*, *r3*, or *r4*), 156PgCad5 spans the *r4* deletion and is therefore *r4*-specific. To differentiate between *r4r4* homozygotes and *r4x* heterozygotes, PCR was performed using 159PgCad5 (5′- CCATAGACCTGGATTCAGGCCA-3′) and 158PgCad3. 159PgCad5 corresponds to the missing nucleotides found in the *r4* deletion and therefore will amplify all alleles except *r4*, allowing for detection of *r4* heterozygotes.

### Greenhouse bioassays

Greenhouse bioassays were used to test the survival of the Bt4R strain on cotton plants [21], [38], [55]. Non-Bt (DP 5415 RR®) and Bt (DP 449 BG®/RR®) plants were grown in 1 gallon pots in a temperature controlled greenhouse (32–35 °C day and 27 °C night) under natural light conditions. Newly emerged neonates (n = 5–15) were placed on 10–14 d old cotton bolls (plant age of 68–106 d) and individual bolls were enclosed in 2″×3″ nylon sacks with a draw string 24 h after infestation. Bolls were infested with either Bt4R or SOM-07. After 35 d, bolls were removed from plants and inspected for exit holes and/or survivors (4^th^ instar larvae, pupae, or adults). Bolls were stored at −20 °C until tested for Cry1Ac by strip or ELISA tests for Cry1Ab/Cry1Ac (Envirologix, Portland, ME). We did two trials, one in September–November, 2009 and the other June–July, 2010. In each trial, 10 Bt plants and 10 non-Bt plants were infested.

### Data Analysis

In diet bioassays, larval survival (%) was calculated as survival on treated diet divided by survival on untreated diet times 100%. We report a maximum of 100% survival, even for a few cases in which survival was higher on treated than untreated diet. For diet bioassays, we estimated dominance (*h*), which varies from 0 for completely recessive resistance to 1 for completely dominant resistance, as described previously [52]. In greenhouse bioassays, survival (%) was calculated as the number of survivors recovered divided by the number of entrance marks times 100%. For each of the two trials, we calculated survival for each pink bollworm strain (resistant and susceptible) on each type of cotton (Bt and non-Bt). We used t-tests to compare survival between strains on Bt cotton and on non-Bt cotton.

## Supporting Information

Figure S1 **Nucleotide and deduced amino acid sequence of the** ***r4*** **cadherin resistance allele.** The cDNA sequence of *PgCad1 r4* allele (accession number JQ279500.1) is shown with deduced ORF below. The location of the *r4* deletion resulting in loss of 5 amino acids from the PgCad1 coding sequence is highlighted in red. Single-base substitutions in *r4 PgCad1* that differ with other cadherin *r* alleles are highlighted in green and amino acid substitutions are highlighted in yellow. The location of primers 156PgCad5 and 158PgCad3 used to PCR amplify and detect the *r4* allele are shown with a single-underline and double-underline, respectively.
(PDF)

Figure S2 **Sequence alignment of** ***PgCad1*** **cadherin alleles.** The CLUSTAL W multiple sequence alignment program was used to align *PgCad1 s* (AY198374.1), *r1* (AY713483.1), *r2* (AY713484.1), and *r3* (AY713485.1) cDNA sequences of with the *r4* cadherin allele. Nucleotides conserved in all of the sequences are marked with “*”. The location of the *r4* deletion from the *PgCad1* is highlighted in red and nucleotide substitutions that are unique to *r4* are highlighted in green. Deletions in *PgCad1* corresponding to *r1*, *r2*, and *r3* from Morin et al. [21] are highlighted in grey. Primers from *r4* reaction 156PgCad5 and 158PgCad3 are shown with a single-underline and double-underline, respectively, whereas the location of 159PgCad5 (which is used with 158PgCad3 in *r4x* reaction) is shown with dashed-underline.
(PDF)

Figure S3 **Sequence alignment of translated** ***PgCad1*** **cadherin alleles.** The CLUSTAL W multiple sequence alignment program was used to align deduced amino acid sequences corresponding to the *PgCad1 s* (AY198374.1), *r1* (AY713483.1), *r2* (AY713484.1), *r3* (AY713485.1), and *r4* cadherin alleles. Residues conserved in all of the sequences are marked with “*” and conservative substitutions are indicated with “:” or “.”. The location of the *r4* deletion resulting in loss of 5 residues from PgCad1 is highlighted in red and amino acid the 14 amino acid substitutions that are unique to *r4* are highlighted in green.
(PDF)
