# Supplementary material for: Similar Genetic Basis of Resistance to Bt Toxin Cry1Ac in Boll-Selected and Diet-Selected Strains of Pink Bollworm
Source: PLoS One. 2012 Apr 18;7(4):e35658. doi: 10.1371/journal.pone.0035658 (PMC3329465; doi:10.1371/journal.pone.0035658)
Supplement: Figure S1 — Nucleotide and deduced amino acid sequence of the r4 cadherin resistance allele. The cDNA sequence of PgCad1 r4 allele (accession number JQ279500.1) is shown with deduced ORF below. The location of the r4 deletion resulting in loss of 5 amino acids from the PgCad1 coding sequence is highlighted in red. Single-base substitutions in r4 PgCad1 that differ with other cadherin r alleles are highlighted in green and amino acid substitutions are highlighted in yellow. The location of primers 156PgCad5 and 158PgCad3 used to PCR amplify and detect the r4 allele are shown with a single-underline and double-underline, respectively. (PDF) [file pone.0035658.s001.pdf]

```
ATGGCGGGTGACGCCTGCATACTGGTGACGGTGCTTCTGACCTTCGCAACATTAGTTTTC    60
 M  A  G  D  A  C  I  L  V  T  V  L  L  T  F  A  T  L  V  F     20

GGGCAAGAAACAGCATCGTCGAGATGTTACTACATGACTGACGCTATTCCGAGAGAACCG   120
 G  Q  E  T  A  S  S  R  C  Y  Y  M  T  D  A  I  P  R  E  P     40

AAACCGGATGATTTGCCTGATTTAGAATGGACTGGTGGATGGACCGACTGGCCTTTGATC   180
 K  P  D  D  L  P  D  L  E  W  T  G  G  W  T  D  W  P  L  I     60

CCGGCTGAGCCAAGAGACGACGTGTGCATAAACGGCTGGTACCCACAACTCACCAGCACT   240
 P  A  E  P  R  D  D  V  C  I  N  G  W  Y  P  Q  L  T  S  T     80

TCTCTCGGCACCATCATCATCCACATGGAAGAGGAAATCGAGGGAGATGTTGCTATCGCT   300
 S  L  G  T  I  I  I  H  M  E  E  E  I  E  G  D  V  A  I  A    100

AAACTTAACTATGATGGTTCTGGAACCCCAGAAATTGTCCAGCCGATGGTTATAGGATCT   360
 K  L  N  Y  D  G  S  G  T  P  E  I  V  Q  P  M  V  I  G  S    120

TTTAACCTGCTAAGTCCAGAGATCCGGAATGAAAACGGGGCGTGGTACCTTTATATAACC   420
 F  N  L  L  S  P  E  I  R  N  E  N  G  A  W  Y  L  Y  I  T    140

AATAGGCAAGATTATGAAACACCAACAATGCGTCGGTATACATTCGACGTCCGAGTGCCA   480
 N  R  Q  D  Y  E  T  P  T  M  R  R  Y  T  F  D  V  R  V  P    160

GACGAGACTCGTGCCGCACGAGTGAGTCTCTCCATCGAAAACATTGACGATAACGACCCT   540
 D  E  T  R  A  A  R  V  S  L  S  I  E  N  I  D  D  N  D  P    180

ATCGTCAGGGTGCTAGACGCTTGCCAAGTGCCGGAATTGGGGGAGCCTCGACTAACAGAC   600
 I  V  R  V  L  D  A  C  Q  V  P  E  L  G  E  P  R  L  T  D    200

TGCGTTTACCAAGTGTCAGACGAAGATGGGAGGCCTAGTATCGAGCCCATGACATTCCGC   660
 C  V  Y  Q  V  S  D  E  D  G  R  P  S  I  E  P  M  T  F  R    220

CTCACATCAGACCGTGAAGACGTACAGATATTCTATGTGGAGCCAGCTCACATTACTGGT   720
 L  T  S  D  R  E  D  V  Q  I  F  Y  V  E  P  A  H  I  T  G    240

GATTGGTTCAACATGCAAATTACTATCGGTATCCTATCAGCGCTTAACTTCGAAAGCAAC   780
 D  W  F  N  M  Q  I  T  I  G  I  L  S  A  L  N  F  E  S  N    260

CCGCTGCACATCTTTCAAATCACTGCTTTGGACTCCTGGCCCAACAACCATACGGTGACG   840
 P  L  H  I  F  Q  I  T  A  L  D  S  W  P  N  N  H  T  V  T    280

GTGATGGTGCAAGTTCAGAATGTGGAGCACCGACCGCCGCGATGGATGGAAATCTCCGCA   900
 V  M  V  Q  V  Q  N  V  E  H  R  P  P  R  W  M  E  I  S  A    300

GTCCAGCAGTTTGACGAGATGACGGAGCAGCAATTCCAGGTGCGCGCCATCGACGGAGAC   960
 V  Q  Q  F  D  E  M  T  E  Q  Q  F  Q  V  R  A  I  D  G  D    320

ACTGGCATCGGGAAAGCTATACACTATACCCTCGAGACAGATGAGGAAGAAGATTTGTTC  1020
 T  G  I  G  K  A  I  H  Y  T  L  E  T  D  E  E  E  D  L  F    340

CTCATCGAAACACTTCCGGGCGGCCATGACGGAGCCATCTTCAGCACTGCCATGATTGAT  1080
 L  I  E  T  L  P  G  G  H  D  G  A  I  F  S  T  A  M  I  D    360

GTGGATAGGCTCCGGCGAGATGTCTTCAGACTGTCCCTGGTGGCATACAAGTACGACAAT  1140
 V  D  R  L  R  R  D  V  F  R  L  S  L  V  A  Y  K  Y  D  N    380

GTGTCCTTCGCCACCCCGACACCCGTCGTGATCATAGTCAATGACATCAACAACAAGAAA  1200
 V  S  F  A  T  P  T  P  V  V  I  I  V  N  D  I  N  N  K  K    400

CCCCAACCGCTGCAAGATGAGTACACAATCTCCATAATGGAAGAAACTCCACTGTCGCTG  1260
 P  Q  P  L  Q  D  E  Y  T  I  S  I  M  E  E  T  P  L  S  L    420
```

```
AATTTTGCTGAACTTTTTGGTTTCTATGATGAAGATTTGATCTACGCACAATTCTTGGCG  1320
 N  F  A  E  L  F  G  F  Y  D  E  D  L  I  Y  A  Q  F  L  A    440

GAAATACAAGGCGAGAACCCTCCAGGCGTAGAGCAAGCGTTTTATATTGCGCCCACCGCA  1380
 E  I  Q  G  E  N  P  P  G  V  E  Q  A  F  Y  I  A  P  T  A    460

GGCTTCCAGAACCAGACATTCGCCACAGGGACTCAAGATCACCGAATGCTGGATTATGAG  1440
 G  F  Q  N  Q  T  F  A  T  G  T  Q  D  H  R  M  L  D  Y  E    480

GATGTTCCTTTCCAAAACATCAAGCTCAAGGTAATAGCAACGGACCGTGACAATACCAAT  1500
 D  V  P  F  Q  N  I  K  L  K  V  I  A  T  D  R  D  N  T  N    500

TTTACTGGAGTCGCGGAAGTCAACGTGAACCTGATTAATTGGAACGACGAGGAGCCGATC  1560
 F  T  G  V  A  E  V  N  V  N  L  I  N  W  N  D  E  E  P  I    520

TTTGAGGAAGACCAGCTCGTTGTCAAGTTCAAGGAGACTGTACCCAAGGACTATCACGTC  1620
 F  E  E  D  Q  L  V  V  K  F  K  E  T  V  P  K  D  Y  H  V    540

GGCAGACTGAGGGCTCACGACCGGGACATAGGAGACAGCGTTGTGCATTCCATCTTGGGA  1680
 G  R  L  R  A  H  D  R  D  I  G  D  S  V  V  H  S  I  L  G    560

AATGCGAATACATTTTTGAGAATCGACGAAGAAACTGGCGACATATACGTAGCTATTGAT  1740
 N  A  N  T  F  L  R  I  D  E  E  T  G  D  I  Y  V  A  I  D    580

GACGCGTTCGATTATCACAGACAGAATGAATTTAACATACAAGTTCGCGCTCAGGGCACC  1800
 D  A  F  D  Y  H  R  Q  N  E  F  N  I  Q  V  R  A  Q  G  T    600

ATGTCGGAGCCAGAGTCCAGGCATACAGCGACTGCTCGGCTGGTCATAGAACTCGAGGAC  1860
 M  S  E  P  E  S  R  H  T  A  T  A  R  L  V  I  E  L  E  D    620

GTCAACGACACACCTCCTACTCTGAGGCTGCCTCGCGTAAGTCCGTCTGTAGAAGAGAAT  1920
 V  N  D  T  P  P  T  L  R  L  P  R  V  S  P  S  V  E  E  N    640

GTGCCAGAGGGCTTTGAAACCAACCGGGAGATAACCGCCACGGACCCTGACACCACAGCA  1980
 V  P  E  G  F  E  T  N  R  E  I  T  A  T  D  P  D  T  T  A    660

TACCTGCAGTTTGAAATAGATTGGGACACATCCTTTGCCACTAAACAGGGGCGTGATACC  2040
 Y  L  Q  F  E  I  D  W  D  T  S  F  A  T  K  Q  G  R  D  T    680

AATCCAATAGAGTTCCACGGATGCGTGGATATAGAAACCATCTTCCCAAACCCAGCCGAC  2100
 N  P  I  E  F  H  G  C  V  D  I  E  T  I  F  P  N  P  A  D    700

ACCAGAGAGGCCGTGGGGCGAGTGGTAGCGAAGGAGATCCGCCATAACGTGACCATCGAT  2160
 T  R  E  A  V  G  R  V  V  A  K  E  I  R  H  N  V  T  I  D    720

TTTGAAGAGTTTGAATTTCTCTACCTCACAGTGAGAGTTCGAGACTTGCACACAGATGAC  2220
 F  E  E  F  E  F  L  Y  L  T  V  R  V  R  D  L  H  T  D  D    740

GGACGAGATTATGATGAATCTACCTTCACGATAATAATAATAGATATGAACGACAACTGG  2280
 G  R  D  Y  D  E  S  T  F  T  I  I  I  I  D  M  N  D  N  W    760

CCTATCTGGGCGTCTGGTTTCCTGAACCAGACCTTCAGTATCCGGGAGCGATCATCTACC  2340
 P  I  W  A  S  G  F  L  N  Q  T  F  S  I  R  E  R  S  S  T    780

GGCGTCGTCATCGGGTCCGTACTCGCTACAGACATTGATGGCCCACTTTACAACCAAGTC  2400
 G  V  V  I  G  S  V  L  A  T  D  I  D  G  P  L  Y  N  Q  V    800

CGGTACACCATTATCCCCCAGGAAGATACTCCTGAAGGTCTAGTCCAGATACACTTCGTT  2460
 R  Y  T  I  I  P  Q  E  D  T  P  E  G  L  V  Q  I  H  F  V    820

ACGGGTCAGATTACAGTTGATGAGAATGGTGCAATCGACGCTGATATTCCACCTCGTTGG  2520
 T  G  Q  I  T  V  D  E  N  G  A  I  D  A  D  I  P  P  R  W    840

CACCTCAACTACACAGTTATAGCCAGCGACAAATGTTCCGAAGAAAATGAAGAGAACTGT  2580
 H  L  N  Y  T  V  I  A  S  D  K  C  S  E  E  N  E  E  N  C    860
```

```
CCCCCGGATCCAGTGTTCTGGGATACTCTGGGCGACAACGTAATTAACATCGTGGACATA  2640
 P  P  D  P  V  F  W  D  T  L  G  D  N  V  I  N  I  V  D  I    880

AACAACAAGGTCCCGGCAGCAGACCTCAGTCGATTCAACGAAACGGTGTACATTTATGAA  2700
 N  N  K  V  P  A  A  D  L  S  R  F  N  E  T  V  Y  I  Y  E    900

AATGCACCCGATTTCACAAACGTGGTCAAGATATACTCCATCGACGAAGACAGAGACGAA  2760
 N  A  P  D  F  T  N  V  V  K  I  Y  S  I  D  E  D  R  D  E    920

ATATATCACACGGTGCGGTACCAGATCAATTATGCTGTGAACCAGCGGCTGCGAGACTTC  2820
 I  Y  H  T  V  R  Y  Q  I  N  Y  A  V  N  Q  R  L  R  D  F    940

TTCGCCGGTGAGGTGTACGTGGAGAACACCAACAATGAGCTCCTGGATCGGGACAGAGGC  2880
 F  A  G  E  V  Y  V  E  N  T  N  N  E  L  L  D  R  D  R  G    960

GAAGACCAACACAGGATATTCATTAACCTCATTGACAACTTTTATAGAGAAGGAGATGGA  2940
 E  D  Q  H  R  I  F  I  N  L  I  D  N  F  Y  R  E  G  D  G    980

AATAGAAATGTAAACACTACAGAGGTGCTGGTGATACTATTAGATGAGAATGACAACGCT  3000
 N  R  N  V  N  T  T  E  V  L  V  I  L  L  D  E  N  D  N  A   1000

CCTGAATTGCCGACTCCAGAAGAGCTGAGTTGGAGCATTTCCGAGAATTTACAAGAGGGT  3060
 P  E  L  P  T  P  E  E  L  S  W  S  I  S  E  N  L  Q  E  G   1020

ATAACACTCGATGGCGAAAGCGATGTGATATACGCACCGGATATAGACGAAGAGGACACG  3120
 I  T  L  D  G  E  S  D  V  I  Y  A  P  D  I  D  E  E  D  T   1040

CCAAACTCTCACGTTGGCTACGCAATCCTGGCCATGACAGTCACCAATAGAGACCTGGAC  3180
 P  N  S  H  V  G  Y  A  I  L  A  M  T  V  T  N  R  D  L  D   1060

ACTGTTCCGAGACTTCTCAACATGCTGTCGCCTAACAACGTAACCGGATTCCTTCAGACA  3240
 T  V  P  R  L  L  N  M  L  S  P  N  N  V  T  G  F  L  Q  T   1080

GCAATGCCTTTGAGAGGATATTGGGGTACTTACGATATAAGTATACTGGCGTTCGACCAC  3300
 A  M  P  L  R  G  Y  W  G  T  Y  D  I  S  I  L  A  F  D  H   1100

GGTATTCCTCAGCAGATATCTCATGAGGTGTATGAACTGGAAATTCGACCTTACAATTAC  3360
 G  I  P  Q  Q  I  S  H  E  V  Y  E  L  E  I  R  P  Y  N  Y   1120

AATCCTCCCCAGTTCGTTTTTCCTGAATCCGGGACGATTCTACGACTGGCTTTGGAACGC  3420
 N  P  P  Q  F  V  F  P  E  S  G  T  I  L  R  L  A  L  E  R   1140

GCAGTGGTAAATAATGTTTTGTCACTTGTAAACGGTGACCCGTTAGACAGGATACAAGCA  3480
 A  V  V  N  N  V  L  S  L  V  N  G  D  P  L  D  R  I  Q  A   1160

ATTGACGACGATGGTCTTGATGCTGGCGTGGTGACTTTCGATATTGTTGGAGATGCTGAT  3540
 I  D  D  D  G  L  D  A  G  V  V  T  F  D  I  V  G  D  A  D   1180

GCATCAAGCTACTTCAGAGTAAATAATGATGGCGACAACTTTGGGACCTTGTTGCTGACA  3600
 A  S  S  Y  F  R  V  N  N  D  G  D  N  F  G  T  L  L  L  T   1200

CAGGCGCTTCCTGAGGAAGGCAAGGAATTTGAGGTTACCATCCGGGCTACAGACGGCGGA  3660
 Q  A  L  P  E  E  G  K  E  F  E  V  T  I  R  A  T  D  G  G   1220

ACGGAACCTCGATCATATTCAACAGACTCCACTATAACAGTGCTCTTCGTTCCGACTTTG  3720
 T  E  P  R  S  Y  S  T  D  S  T  I  T  V  L  F  V  P  T  L   1240

GGTGATCCGATCTTTCAAGATAACACTTACTCAGTAGCATTCTTTGAAAAAGAGGTTGGC  3780
 G  D  P  I  F  Q  D  N  T  Y  S  V  A  F  F  E  K  E  V  G   1260

TTGACTGAGAGGTTCTCGCTCCCACATGCAGAGGACCCTAAGAACAAACTCTGCACTGAC  3840
 L  T  E  R  F  S  L  P  H  A  E  D  P  K  N  K  L  C  T  D   1280

GACTGTCACGATATTTACTACAGGATCTTTGGTGGTGTGGATTACGAGCCATTTGACCTG  3900
 D  C  H  D  I  Y  Y  R  I  F  G  G  V  D  Y  E  P  F  D  L   1300
```

```
GACCCGGTGACGAACGTGATCTTCCTGAAATCAGAACTAGACCGGGAGACCACTGCCACG  3960
 D  P  V  T  N  V  I  F  L  K  S  E  L  D  R  E  T  T  A  T   1320

CATGTGGTGCAAGTGGCAGCCAGTAATTCGCCCACAGGAGGCGGAATACCACTCCCTGGG  4020
 H  V  V  Q  V  A  A  S  N  S  P  T  G  G  G  I  P  L  P  G   1340

TCTCTTCTCACCGTCACTGTCACTGTACGAGAAGCGGATCCACGGCCTGTGTTCGAGCAG  4080
 S  L  L  T  V  T  V  T  V  R  E  A  D  P  R  P  V  F  E  Q   1360

CGTCTGTACACGGCTGGCATTTCCACTTCCGATAACATCAACAGGGAACTACTCACCGTT  4140
 R  L  Y  T  A  G  I  S  T  S  D  N  I  N  R  E  L  L  T  V   1380

CGTGCAACTCATTCCGAAAACGCACAATTGACATATACCATCGAAGACGGTTCTATGGCG  4200
 R  A  T  H  S  E  N  A  Q  L  T  Y  T  I  E  D  G  S  M  A   1400

GTGGACTCCACTCTGGAAGCCGTCAAGGACTCGGCGTTCCATCTGAACGCGCAGACCGGC  4260
 V  D  S  T  L  E  A  V  K  D  S  A  F  H  L  N  A  Q  T  G   1420

GTCCTCATACTGAGGATACAACCTACTGCCAGCATGCAGGGCATGTTCGAGTTCAACGTC  4320
 V  L  I  L  R  I  Q  P  T  A  S  M  Q  G  M  F  E  F  N  V   1440

ATCGCTACTGATCCAGATGAGAAGACAGATACGGCAGAGGTGAAAGTCTACCTCATTTCA  4380
 I  A  T  D  P  D  E  K  T  D  T  A  E  V  K  V  Y  L  I  S   1460

TCCCAAAATAGGGTGTCCTTCATATTCCTGAACGATGTGGAGACGGTTGAGAGTAACAGA  4440
 S  Q  N  R  V  S  F  I  F  L  N  D  V  E  T  V  E  S  N  R   1480

GACTTTATCGCAGAAACGTTCAGCGTTGGCTTCAACATGACCTGCAATATAGATCAGGTG  4500
 D  F  I  A  E  T  F  S  V  G  F  N  M  T  C  N  I  D  Q  V   1500

CTGCCGGGCACCAACGGCGCCGGGGTGATTCAGGAGGCCATGGCGGAAGTCCATGCTCAC  4560
 L  P  G  T  N  G  A  G  V  I  Q  E  A  M  A  E  V  H  A  H   1520

TTCATACAGGATAACATCCCTGTGAGCGCCGACAGTATTGAAGAGCTTCGCAGTGACACT  4620
 F  I  Q  D  N  I  P  V  S  A  D  S  I  E  E  L  R  S  D  T   1540

CAGCTGCTGCGCTCCGTCCAAGGTGTGTTGAACCAGCGGCTGTTGGTCCTGAACGACCTG  4680
 Q  L  L  R  S  V  Q  G  V  L  N  Q  R  L  L  V  L  N  D  L   1560

GTGACGGGGGTCAGCCCTGATCTCGGCACTGCCGGCGTGCAGATCACCATCTATGTGCTA  4740
 V  T  G  V  S  P  D  L  G  T  A  G  V  Q  I  T  I  Y  V  L   1580

GCCGGGTTGTCAGCCATCCTTGCCTTCCTGTGCCTTATTCTGCTCATCACATTCATCGTG  4800
 A  G  L  S  A  I  L  A  F  L  C  L  I  L  L  I  T  F  I  V   1600

AGGACCCGAGCTCTGAACCGCCGTTTGGAAGCACTGTCGATGACGAAATACGGCTCGGTG  4860
 R  T  R  A  L  N  R  R  L  E  A  L  S  M  T  K  Y  G  S  V   1620

GATTCGGGGCTGAACCGAGTGGGGATAGCGGCCCCAGGAACCAACAAACACGCCATCGAA  4920
 D  S  G  L  N  R  V  G  I  A  A  P  G  T  N  K  H  A  I  E   1640

GGCTCCAACCCCATCTGGAACGAGCAGATCAAGGCCCCGGACTTCGATGCCATCAGTGAC  4980
 G  S  N  P  I  W  N  E  Q  I  K  A  P  D  F  D  A  I  S  D   1660

ACATCTGACGAGTCTGATCTGATCGGCATCGAGGATCTACCACAATTCAAGAGCGACTAT  5040
 T  S  D  E  S  D  L  I  G  I  E  D  L  P  Q  F  K  S  D  Y   1680

TTCCCGCCTGAGGACTCGGAATCCGCTCACGCCGCCTTTAGCGACCGCACGCCACGCGGG  5100
 F  P  P  E  D  S  E  S  A  H  A  A  F  S  D  R  T  P  R  G   1700

AACGATGCGCCTATTGCACACAGTAGCAACAACTTCGGTTTCAACACCAGCCCTTTTAGC  5160
 N  D  A  P  I  A  H  S  S  N  N  F  G  F  N  T  S  P  F  S   1720

GCGGAGTTCACTAACAGGCGCATGCGACCATAG                             5193
 A  E  F  T  N  R  R  M  R  P  -                              1730
```
