# Supplementary material for: Similar Genetic Basis of Resistance to Bt Toxin Cry1Ac in Boll-Selected and Diet-Selected Strains of Pink Bollworm
Source: PLoS One. 2012 Apr 18;7(4):e35658. doi: 10.1371/journal.pone.0035658 (PMC3329465; doi:10.1371/journal.pone.0035658)
Supplement: Figure S2 — Sequence alignment of PgCad1 cadherin alleles. The CLUSTAL W multiple sequence alignment program was used to align PgCad1 s (AY198374.1), r1 (AY713483.1), r2 (AY713484.1), and r3 (AY713485.1) cDNA sequences of with the r4 cadherin allele. Nucleotides conserved in all of the sequences are marked with “*”. The location of the r4 deletion from the PgCad1 is highlighted in red and nucleotide substitutions that are unique to r4 are highlighted in green. Deletions in PgCad1 corresponding to r1, r2, and r3 from Morin et al. [21] are highlighted in grey. Primers from r4 reaction 156PgCad5 and 158PgCad3 are shown with a single-underline and double-underline, respectively, whereas the location of 159PgCad5 (which is used with 158PgCad3 in r4x reaction) is shown with dashed-underline. (PDF) [file pone.0035658.s002.pdf]

```
CLUSTAL 2.1 multiple sequence alignment

PgCad1_r4       ATGGCGGGTGACGCCTGCATACTGGTGACGGTGCTTCTGACCTTCGCAAC 50
AY713484.1_r2   ATGGCGGGTGACGCCTGCATACTGGTGACGGTGCTTCTGATCTTCGCAAT 50
AY198374.1_s    ATGGCGGGTGACGCCTGCATACTGGTGACGGTGCTTCTGACCTTCGCAAC 50
AY713483.1_r1   ATGGCGGGTGACGCCTGCATACTGGTGACGGTGCTTCTCACCTTCGCAAC 50
AY713485.1_r3   ATGGCGGGTGACGCCTGCATACTGGTGACGGTGCTTCTCACCTTCGCAAC 50
                ************************************** * ******** 

PgCad1_r4       ATTAGTTTTCGGGCAAGAAACAGCATCGTCGAGATGTTACTACATGACTG 100
AY713484.1_r2   ATCAGTTTTCGGGCAAGAAACAGCATCGTCGAGATGTTACTACATGACTG 100
AY198374.1_s    ATCAGTTTTCGGGCAAGAAACAACATCGTCGAGATGTTACTACATGACTG 100
AY713483.1_r1   ATCAGTTTTCGGGCAAGAAACAACATCGTCGAGATGTTACTACATGACTG 100
AY713485.1_r3   ATCAGTTTTCGGGCAAGAAACAACATCGTCGAGATGTTACTACATGACTG 100
                ** ******************* ***************************

PgCad1_r4       ACGCTATTCCGAGAGAACCGAAACCGGATGATTTGCCTGATTTAGAATGG 150
AY713484.1_r2   ACGCTATTCCGAGAGAACCGAAACCGGATGATTTGCCTGATTTAGAATGG 150
AY198374.1_s    ACGCTATTCCGAGAGAACCGAAACCGGATGATTTGCCTGATTTAGAATGG 150
AY713483.1_r1   ACGCTATTCCGAGAGAACCGAAACCGGATGATTTGCCTGATTTAGAATGG 150
AY713485.1_r3   ACGCTATTCCGAGGGAACCGAAACCGGATGATTTGCCTGACTTAGAATGG 150
                ************* ************************** *********

PgCad1_r4       ACTGGTGGATGGACCGACTGGCCTTTGATCCCGGCTGAGCCAAGAGACGA 200
AY713484.1_r2   ACTGGTGGATGGACCGACTGGCCTTTGATCCCGGCTGAGCCAAGAGACGA 200
AY198374.1_s    ACTGGTGGATGGACCGACTGGCCTTTGATCCCGGCTGAGCCAAGAGACGA 200
AY713483.1_r1   ACTGGTGGATGGACCGACTGGCCTTTGATCCCGGCTGAGCCAAGAGACGA 200
AY713485.1_r3   ACTGGTGGATGGACCGACTGGCCTTTGATCCCGGCTGAGCCAAGAGACGA 200
                **************************************************

PgCad1_r4       CGTGTGCATAAACGGCTGGTACCCACAACTCACCAGCACTTCTCTCGGCA 250
AY713484.1_r2   CGTGTGCATAAACGGCTGGTACCCACAACTCACCAGCACTTCTCTCGGCA 250
AY198374.1_s    CGTGTGCATAAACGGCTGGTACCCACAACTCACCAGCACTTCTCTCGGCA 250
AY713483.1_r1   CGTGTGCATAAACGGCTGGTACCCACAACTCACCAGCACTTCTCTCGGCA 250
AY713485.1_r3   CGTGTGCATAAACGGCTGGTACCCACAACTCACCAGCACTTCTCTCGGCA 250
                **************************************************

PgCad1_r4       CCATCATCATCCACATGGAAGAGGAAATCGAGGGAGATGTTGCTATCGCT 300
AY713484.1_r2   CCATCATCATCCACATGGAAGAGGAGATCGAGGGAGATGTTGCTATCGCT 300
AY198374.1_s    CCATCATCATCCACATGGAAGAGGAGATCGAGGGAGATGTTGCTATCGCT 300
AY713483.1_r1   CCATCATCATCCACATGGAAGAGGAGATCGAGGGAGATGTTGCTATCGCT 300
AY713485.1_r3   CCATCATCATCCACATGGAAGAGGAGATCGAGGGAGATGTTGCTATCGCT 300
                ************************* ************************

PgCad1_r4       AAACTTAACTATGATGGTTCTGGAACCCCAGAAATTGTCCAGCCGATGGT 350
AY713484.1_r2   AAACTTAACTATGATGGTTCTGGAACCCCAGAAATTGTCCAGCCGATGGT 350
AY198374.1_s    AAACTTAACTATGATGGTTCTGGAACCCCAGAAATTGTCCAGCCGATGGT 350
AY713483.1_r1   AAACTTAACTATGATGGTTCTGGAACCCCAGAAATTGTCCAGCCGATGGT 350
AY713485.1_r3   AAACTTAACTATGATGGTTCTGGAACCCCAGAAATTGTCCAGCCGATGGT 350
                **************************************************

PgCad1_r4       TATAGGATCTTTTAACCTGCTAAGTCCAGAGATCCGGAATGAAAACGGGG 400
AY713484.1_r2   TATAGGATCTTTTAACCTGCTAAGTCCAGAGATCCGGAATGAAAACGGGG 400
AY198374.1_s    TATAGGATCTTTTAACCTGCTAAGTCCAGAGATCCGGAATGAAAACGGGG 400
AY713483.1_r1   TATAGGATCTTTTAACCTGCTAAGTCCAGAGATCCGGAATGAAAACGGGG 400
AY713485.1_r3   TATAGGATCTTTTAACCTGCTAAGTCCAGAGATCCGGAATGAAAACGGGG 400
                **************************************************

PgCad1_r4       CGTGGTACCTTTATATAACCAATAGGCAAGATTATGAAACACCAACAATG 450
AY713484.1_r2   CGTGGTACCTTTATATAACCAATAGGCAAGATTATGAAACACCAACAATG 450
AY198374.1_s    CGTGGTACCTTTATATAACCAATAGGCAAGATTATGAAACACCAACAATG 450
AY713483.1_r1   CGTGGTACCTTTATATAACCAATAGGCAAGATTATGAAACACCAACAATG 450
AY713485.1_r3   CGTGGTACCTTTATATAACCAATAGGCAAGATTATGAAACACCAACAATG 450
                **************************************************
```

```
PgCad1_r4       CGTCGGTATACATTCGACGTCCGAGTGCCAGACGAGACTCGTGCCGCACG 500
AY713484.1_r2   CGTCGGTATACATTCGACGTCCGAGTGCCAGACGAGACTCGTGCGGCACG 500
AY198374.1_s    CGTCGGTATACATTCGACGTCCGAGTGCCAGACGAGACTCGTGCGGCACG 500
AY713483.1_r1   CGTCGGTATACATTCGACGTCCGAGTGCCAGACGAGACTCGTGCGGCACG 500
AY713485.1_r3   CGTCGGTATACATTCGACGTCCGAGTGCCAGACGAGACTCGTGCGGCACG 500
                ******************************************** *****

PgCad1_r4       AGTGAGTCTCTCCATCGAAAACATTGACGATAACGACCCTATCGTCAGGG 550
AY713484.1_r2   AGTGAGTCTGTCCATCGAAAACATTGACGATAACGACCCTATCGTCAGGG 550
AY198374.1_s    AGTGAGTCTGTCCATCGAAAACATTGACGATAACGACCCTATCGTCAGGG 550
AY713483.1_r1   AGTGAGTCTCTCCATCGAAAACATTGACGATAACGACCCTATCGTCAGGG 550
AY713485.1_r3   AGTGAGTCTGTCCATCGAAAACATTGACGATAACGACCCTATCGTCAGGG 550
                ********* ****************************************

PgCad1_r4       TGCTAGACGCTTGCCAAGTGCCGGAATTGGGGGAGCCTCGACTAACAGAC 600
AY713484.1_r2   TGCTAGACGCTTGCCAAGTGCCGGAATTGGGGGAGCCTCGACTAACAGAC 600
AY198374.1_s    TGCTAGACGCTTGCCAAGTGCCGGAATTGGGGGAGCCTCGACTAACAGAC 600
AY713483.1_r1   TGCTAGACGCTTGCCAAGTGCCGGAATTGGGGGAGCCTCGACTAACTGAC 600
AY713485.1_r3   TGCTAGACGCTTGCCAAGTGCCGGAATTGGGGGAGCCTCGACTAACAGAC 600
                ********************************************** ***

PgCad1_r4       TGCGTTTACCAAGTGTCAGACGAAGATGGGAGGCCTAGTATCGAGCCCAT 650
AY713484.1_r2   TGCGTTTACCAAGTGTCAGACGAAGATGGGAGGCTTAGTATCGAGCCCAT 650
AY198374.1_s    TGCGTTTACCAAGTGTCAGACGAAGATGGGAGGCTTAGTATCGAGCCCAT 650
AY713483.1_r1   TGCGTTTACCAAGTGTCAGACGAAGATGGGAGGCTTAGTATCGAGCCCAT 650
AY713485.1_r3   TGCGTTTACCAAGTGTCAGACGAAGATGGGAGGCTTAGTATCGAGCCCAT 650
                ********************************** ***************

PgCad1_r4       GACATTCCGCCTCACATCAGACCGTGAAGACGTACAGATATTCTATGTGG 700
AY713484.1_r2   GACATTCCGCCTCACATCAGACCGTGAAGACGTACAGATATTCTATGTGG 700
AY198374.1_s    GACATTCCGCCTCACATCAGACCGTGAAGACGTACAGATATTCTATGTGG 700
AY713483.1_r1   GACATTCCGCCTCACATCAGACCGTGAAGACGTACAGATATTCTACGTGG 700
AY713485.1_r3   GACATTCCGCCTCACATCAGACCGTGAAGACGTACAGATATTCTATGTGG 700
                ********************************************* ****

PgCad1_r4       AGCCAGCTCACATTACTGGTGATTGGTTCAACATGCAAATTACTATCGGT 750
AY713484.1_r2   AGCCAGCTCACATTACTGGTGATTGGTTCAACATGCAAATTACTATCGGT 750
AY198374.1_s    AGCCAGCTCACATTACTGGTGATTGGTTCAACATGCAAATTACTATCGGT 750
AY713483.1_r1   AGCCAGCTCACATTACTGGAGATTGGTTCAACATGCAAATTACTATCGGT 750
AY713485.1_r3   AGCCAGCTCACATTACTGGTGATTGGTTCAACATGCAAATTACTATCGGT 750
                ******************* ******************************

PgCad1_r4       ATCCTATCAGCGCTTAACTTCGAAAGCAACCCGCTGCACATCTTTCAAAT 800
AY713484.1_r2   ATCCTATCAGCGCTTAACTTCGAAAGCAACCCGCTGCACATCTTTCAAAT 800
AY198374.1_s    ATCCTATCAGCGCTTAACTTCGAAAGCAACCCGCTGCACATCTTTCAAAT 800
AY713483.1_r1   ATCCTATCAGCGCTTAACTTCGAAAGCAACCCGCTTCACATCTTTCAAAT 800
AY713485.1_r3   ATCCTATCAGCGCTTAACTTCGAAAGCAACCCGCTGCACATCTTTCAAAT 800
                *********************************** **************

PgCad1_r4       CACTGCTTTGGACTCCTGGCCCAACAACCATACGGTGACGGTGATGGTGC 850
AY713484.1_r2   CACTGCTTTGGACTCCTGGCCCAACAACCATACGGTGACGGTGATGGTGC 850
AY198374.1_s    CACTGCTTTGGACTCCTGGCCCAACAACCATACGGTGACGGTGATGGTGC 850
AY713483.1_r1   CACTGCTTTGGACTCCTGGCCCAACAACCATACGGTGACGGTGATGGTGC 850
AY713485.1_r3   CACTGCTTTGGACTCCTGGCCCAACAACCATACGGTGACGGTGATGGTGC 850
                **************************************************

PgCad1_r4       AAGTTCAGAATGTGGAGCACCGACCGCCGCGATGGATGGAAATCTCCGCA 900
AY713484.1_r2   AAGTCCAGAATGTGGAGCACCGACCGCCGCGATGGATGGAAATCTTCGCA 900
AY198374.1_s    AAGTTCAGAATGTGGAGCACCGACCGCCGCGATGGATGGAAATCTTCGCA 900
AY713483.1_r1   AAGTCCAGAATGTGGAGCACCGACCGCCGCGATGGATGGAAATCTTCGCA 900
AY713485.1_r3   AAGTCCAGAATGTGGAACACCGACCGCCGCGATGGATGGAAATCTTCGCA 900
                **** *********** **************************** ****

PgCad1_r4       GTCCAGCAGTTTGACGAGATGACGGAGCAGCAATTCCAGGTGCGCGCCAT 950
AY713484.1_r2   GTCCAGCAGTTTGACGAGATGACGGAGCAGCAATTCCAGGTGCGCGCCAT 950
AY198374.1_s    GTCCAGCAGTTTGACGAGATGACGGAGCAGCAATTCCAGGTGCGCGCCAT 950
AY713483.1_r1   GTCCAGCAGTTTGACGAGATGACGGAGCAGCAATTCCAGGTGCGCGCCAT 950
AY713485.1_r3   GTCCAGCAGTTTGACGAGATGACGGAGCAGCAATTCCAGGTGCGCGCCAT 950
                **************************************************
```

```
PgCad1_r4       CGACGGAGACACTGGCATCGGGAAAGCTATACACTATACCCTCGAGACAG 1000
AY713484.1_r2   CGACGGAGACACTGGCATCGGGAAAGCTATACACTATACCCTCGAGACAG 1000
AY198374.1_s    CGACGGAGACACTGGCATCGGGAAAGCTATACACTATACCCTCGAGACAG 1000
AY713483.1_r1   CGACGGAGACACTGGCATCGGGAAAGCTATACACTATACCCTCGAGACAG 1000
AY713485.1_r3   CGACGGAGACACTGGCATCGGGAAAGCTATACACTATACCCTCGAGACAG 1000
                **************************************************

PgCad1_r4       ATGAGGAAGAAGATTTGTTCCTCATCGAAACACTTCCGGGCGGCCATGAC 1050
AY713484.1_r2   ATGAGGAAGAAGATTTGTTCTTCATCGAAACACTTCCGGGCGGCCATGAC 1050
AY198374.1_s    ATGAGGAAGAAGATTTGTTCTTCATCGAAACACTTCCGGGCGGCCATGAC 1050
AY713483.1_r1   ATGAGGAAGAAGATTTGTTCTTCATCGAAACACTTCCGGGCGGCCATGAC 1050
AY713485.1_r3   ATGAGGAAGAAGATTTGTTCTTCATCGAAACACTTCCGGGCGGCCATGAC 1050
                ******************** *****************************

PgCad1_r4       GGAGCCATCTTCAGCACTGCCATGATTGATGTGGATAGGCTCCGGCGAGA 1100
AY713484.1_r2   GGAGCCATCTTCAGCACTGCCATGATTGATGTGGATAGGCTCCGGCGAGA 1100
AY198374.1_s    GGAGCCATCTTCAGCACTGCCATGATTGATGTGGATAGGCTCCGGCGAGA 1100
AY713483.1_r1   GGAGCCATCTTCAGCACTGCCATGATTGATGTGGATAGGCTCCGGCGAGA 1100
AY713485.1_r3   GGAGCCATCTTCAGCACTGCCATGATTGATGTGGATAGGCTCCGGCGAGA 1100
                **************************************************

PgCad1_r4       TGTCTTCAGACTGTCCCTGGTGGCATACAAGTACGACAATGTGTCCTTCG 1150
AY713484.1_r2   TGTCTTCAGACTGTCCCTGGTGGCATACAAGTACGACAATGTGTCCTTCG 1150
AY198374.1_s    TGTCTTCAGACTGTCCCTGGTGGCATACAAGTACGACAATGTGTCCTTCG 1150
AY713483.1_r1   TGTCTTCAGACTGTCCCTGGTGGCATACAAGTACGACAATGTGTCCTTCG 1150
AY713485.1_r3   TGTCTTCAGGCTGTCCCTGGTGGCATACAAGTACGACAATGTGTCCTTCG 1150
                ********* ****************************************

PgCad1_r4       CCACCCCGACACCCGTCGTGATCATAGTCAATGACATCAACAACAAGAAA 1200
AY713484.1_r2   CCACCCCGACACCCGTCGTGATCATAGTCAACGACATCAACAACAAGCAA 1200
AY198374.1_s    CCACCCCGACACCCGTCGTGATCATAGTCAATGACATCAACAACAAGAAA 1200
AY713483.1_r1   CCACCCCGACACCCGTCGTGATCATAGTTAACGACATCAACAACAAGAAA 1200
AY713485.1_r3   CCACCCCGACACCCGTCGTGATCATAGTCAATGACATCAACAACAAGAAA 1200
                **************************** ** *************** **

PgCad1_r4       CCCCAACCGCTGCAAGATGAGTACACAATCTCCATAATGGAAGAAACTCC 1250
AY713484.1_r2   CCCCAACCGCTGCAAGATGAGTACACAATCTCCATAATGGAAGAAACTCC 1250
AY198374.1_s    CCCCAACCGCTGCAAGATGAGTACACAATCTCCATAATGGAAGAAACTCC 1250
AY713483.1_r1   CCCCAACCGCTGCAAGATGAGTACACAATCTCCATAATGGAAGAAACTCC 1250
AY713485.1_r3   CCCCAACCGCTGCAAGATGAGTACACAATCTCCATAATGGAAGAAACTCC 1250
                **************************************************

PgCad1_r4       ACTGTCGCTGAATTTTGCTGAACTTTTTGGTTTCTATGATGAAGATTTGA 1300
AY713484.1_r2   ACTGTCGCTGAATTTTGCTGAACTTTTTGGTTTCTATGATGAAGATTTGA 1300
AY198374.1_s    ACTGTCGCTGAATTTTGCTGAACTTTTTGGTTTCTATGATGAAGATTTGA 1300
AY713483.1_r1   ACTGTCGCTGAATTTTGCTGAACTTTTTGGTTTCTATGATGAAGATTTGA 1300
AY713485.1_r3   ACTGTCGCTGAATTTTGCTGAACTTTTTGGTTTCTATGATGAAGATTTGA 1300
                **************************************************

PgCad1_r4       TCTACGCACAATTCTTGGCGGAAATACAAGGCGAGAACCCTCCAGGCGTA 1350
AY713484.1_r2   TCTACGCACAATTCTTGGTGGAAATACAAGGCGAGAACCCTCCAGGCGTA 1350
AY198374.1_s    TCTACGCACAATTCTTGGTGGAAATACAAGGCGAGAACCCTCCAGGCGTA 1350
AY713483.1_r1   TCTACGCACAATTCTTGGTGGAAATACAAGGCGAGAACCCTCCAGGCGTA 1350
AY713485.1_r3   TCTACGCACAATTCTTGGTGGAAATACAAGGCGAGAACCCTCCAGGCGTA 1350
                ****************** *******************************

PgCad1_r4       GAGCAAGCGTTTTATATTGCGCCCACCGCAGGCTTCCAGAACCAGACATT 1400
AY713484.1_r2   GAGCAAGCGTTTTATATTGCGCCCACCGCAGGCTTCCAGAACCAGACATT 1400
AY198374.1_s    GAGCAAGCGTTTTATATTGCGCCCACCGCAGGCTTCCAGAACCAGACATT 1400
AY713483.1_r1   GAGCAAGCGTTTTATATTGCGCCCACCGCAGGCTTCCAGAACCAGACATT 1400
AY713485.1_r3   GAGCAAGCGTTTTATATTGCGCCCACCGCAGGCTTCCAGAACCAGACATT 1400
                **************************************************

PgCad1_r4       CGCCACAGGGACTCAAGATCACCGAATGCTGGATTATGAGGATGTTCCTT 1450
AY713484.1_r2   CGCCATAGGGACTCAAGATCACCGAATGCTGGATTATGAGGATGTTCCTT 1450
AY198374.1_s    CGCCATAGGGACTCAAGATCACCGAATGCTGGATTATGAGGATGTTCCTT 1450
AY713483.1_r1   CGCCATAGGGACTCAAGATCACCGAATGCTGGATTATGAGGATGTTCCTT 1450
AY713485.1_r3   CGCCATAGGGACTCAAGATCACCGAATGCTGGATTATGAGGATGTTCCTT 1450
                ***** ********************************************
```

```
PgCad1_r4       TCCAAAACATCAAGCTCAAGGTAATAGCAACGGACCGTGACAATACCAAT 1500
AY713484.1_r2   TCCAAAACATCAAGCTCAAGGTAATAGCAACGGACCGTGACAATACCAAT 1500
AY198374.1_s    TCCAAAACATCAAGCTCAAGGTAATAGCAACGGACCGTGACAATACCAAT 1500
AY713483.1_r1   TCCAAAACATCAAGCTCAAGGTAATAGCAACGGACCGTGACAATACCAAT 1500
AY713485.1_r3   TCCAAAACATCAAGCTCAAGGTAATAGCAACGGACCGTGACAATACCAAT 1500
                **************************************************

PgCad1_r4       TTTACTGGAGTCGCGGAAGTCAACGTGAACCTGATTAATTGGAACGACGA 1550
AY713484.1_r2   TTCACTGGAGTGGCGGAAGTCAACGTGAATCTGATTAATTGGAACGACGA 1550
AY198374.1_s    TTTACTGGAGTCGCGGAAGTCAACGTGAACCTGATTAATTGGAACGACGA 1550
AY713483.1_r1   TTTACTGGAGTCGCGGAAGTCAACGTGAATCTGATTAATTGGAACGACGA 1550
AY713485.1_r3   TTTACTGGAGTCGCGGAAGTCAACGTGAACCTGATTAATTGGAACGACGA 1550
                ** ******** ***************** ********************

PgCad1_r4       GGAGCCGATCTTTGAGGAAGACCAGCTCGTTGTCAAGTTCAAGGAGACTG 1600
AY713484.1_r2   GGAGCCGATCTTTGAGGAGGACCAGCTGGTTGTCAAGTTCAAGGAGACTG 1600
AY198374.1_s    GGAGCCGATCTTTGAGGAAGACCAGCTCGTTGTCAAGTTCAAGGAGACTG 1600
AY713483.1_r1   GGAGCCGATCTTTGAGGAGGACCAGCTGGTTGTCAAGTTCAAGGAGACTG 1600
AY713485.1_r3   GGAGCCGATCTTTGAGGAAGACCAGCTCGTTGTCAAGTTCAAGGAGACTG 1600
                ****************** ******** **********************

PgCad1_r4       TACCCAAGGACTATCACGTCGGCAGACTGAGGGCTCACGACCGGGACATA 1650
AY713484.1_r2   TACCCAAGGACTATCACGTCGGCAGACTGAGGGCTCACGACCGGGACATA 1650
AY198374.1_s    TACCCAAGGACTATCACGTCGGCAGACTGAGGGCTCACGACCGGGACATA 1650
AY713483.1_r1   TACCCAAGGACTATCACGTCGGCAGACTGAGGGCTCACGACCGGGACATA 1650
AY713485.1_r3   TACCCAAGGACTATCACGTCGGCAGACTGAGGGCTCACGACCGGGACATA 1650
                **************************************************

PgCad1_r4       GGAGACAGCGTTGTGCATTCCATCTTGGGAAATGCGAATACATTTTTGAG 1700
AY713484.1_r2   GGAGACAGCGTTGTGCATTCCATCTTGGGAAATGCGAATACATTTTTGAG 1700
AY198374.1_s    GGAGACAGCGTTGTGCATTCCATCTTGGGAAATGCGAATACATTTTTGAG 1700
AY713483.1_r1   GGAGACAGCGTTGTGCATTCCATCTTGGGAAATGCGAATACATTTTTGAG 1700
AY713485.1_r3   GGAGACAGCGTTGTGCATTCCATCTTGGGAAATGCGAATACATTTTTGAG 1700
                **************************************************

PgCad1_r4       AATCGACGAAGAAACTGGCGACATATACGTAGCTATTGATGACGCGTTCG 1750
AY713484.1_r2   AATCGACGAAGAAACTGGCGACATCTACGTAGCTATTGATGACGCGTTCG 1750
AY198374.1_s    AATCGACGAAGAAACTGGCGACATATACGTAGCTATTGATGACGCGTTCG 1750
AY713483.1_r1   AATCGACGAAGAAACTGGCGACATCTACGTAGCTATTGATGACGCGTTCG 1750
AY713485.1_r3   AATCGACGAAGAAACTGGCGACATATACGTAGCTATTGATGACGCGTTCG 1750
                ************************ *************************

PgCad1_r4       ATTATCACAGACAGAATGAATTTAACATACAAGTTCGCGCTCAGGGCACC 1800
AY713484.1_r2   ATTATCACAGACAGAATGAATTTAACATACAAGTTCGCGCTCAGGACACC 1800
AY198374.1_s    ATTATCACAGACAGAATGAATTTAACATACAAGTTCGCGCTCAGGACACC 1800
AY713483.1_r1   ATTATCACAGACAGAATGAATTTAACATACAAGTTCGCGCTCAGGACACC 1800
AY713485.1_r3   ATTATCACAGACAGAATGAATTTAACATACAAGTTCGCGCTCAGGACACC 1800
                ********************************************* ****

PgCad1_r4       ATGTCGGAGCCAGAGTCCAGGCATACAGCGACTGCTCGGCTGGTCATAGA 1850
AY713484.1_r2   ATGTCGGAGCCAGAGTCCAGGCATACAGCGACTGCTCAGCTGGTCATAGA 1850
AY198374.1_s    ATGTCGGAGCCAGAGTCCAGGCATACAGCGACTGCTCAGCTGGTCATAGA 1850
AY713483.1_r1   ATGTCGGAGCCAGAGTCCAGGCATACAGCGACTGCTCAGCTGGTCATAGA 1850
AY713485.1_r3   ATGTCGGAGCCAGAGTCCAGGCATACAGCGACTGCTCAGCTGGTCATAGA 1850
                ************************************* ************

PgCad1_r4       ACTCGAGGACGTCAACGACACACCTCCTACTCTGAGGCTGCCTCGCGTAA 1900
AY713484.1_r2   ACTCGAGGACGTCAACAACACACCTCCCACTCTGAGGCTGCCTCGCGTAA 1900
AY198374.1_s    ACTCGAGGACGTCAACAACACACCTCCTACTCTGAGGCTGCCTCGCGTAA 1900
AY713483.1_r1   ACTCGAGGACGTCAACAACACACCTCCTACTCTGAGGCTGCCTCGCGTAA 1900
AY713485.1_r3   ACTCGAGGACGTCAACAACACACCTCCTACTCTGAGGCTGCCTCGCGTAA 1900
                **************** ********** **********************

PgCad1_r4       GTCCGTCTGTAGAAGAGAATGTGCCAGAGGGCTTTGAAACCAACCGGGAG 1950
AY713484.1_r2   GTCCGTCTGTAGAAGAGAATGTGCCAGAGGGCTTTGAAATCAACCGGGAG 1950
AY198374.1_s    GTCCGTCTGTAGAAGAGAATGTGCCAGAGGGCTTTGAAATCAACCGGGAG 1950
AY713483.1_r1   GTCCGTCTGTAGAAGAGAATGTGCCAGAGGGCTTTGAAGTCAACCGGGAG 1950
AY713485.1_r3   GTCCGTCTGTAGAAGAGAATGTGCCAGAGGGCTTTGAAATCAACCGGGAG 1950
                **************************************  **********
```

```
PgCad1_r4       ATAACCGCCACGGACCCTGACACCACAGCATACCTGCAGTTTGAAATAGA 2000
AY713484.1_r2   ATAACCGCCACGGACCCTGACACCACAGCATACCTGCAGTTTGAAATAGA 2000
AY198374.1_s    ATAACCGCCACGGACCCTGACACCACAGCATACCTGCAGTTTGAAATAGA 2000
AY713483.1_r1   ATAACCGCCACGGACCCTGACACCACAGCATACCTGCAGTTTGAAATAGA 2000
AY713485.1_r3   ATAACCGCCACGGACCCTGACACCACAGCATACCTGCAGTTTGAAATAGA 2000
                **************************************************

PgCad1_r4       TTGGGACACATCCTTTGCCACTAAACAGGGGCGTGATACCAATCCAATAG 2050
AY713484.1_r2   TTGGGACACATCCTTTGCCACTAAACAGGGGCGTGATACCAATCCAATAG 2050
AY198374.1_s    TTGGGACACATCCTTTGCCACTAAACAGGGGCGTGATACCAATCCAATAG 2050
AY713483.1_r1   TTGGGACACATCCTTTGCCACTAAACAGGGGCGTGATACCAATCCAATAG 2050
AY713485.1_r3   TTGGGACACATCCTTTGCCACTAAACAGGGGCGTGATACCAATCCAATAG 2050
                **************************************************

PgCad1_r4       AGTTCCACGGATGCGTGGATATAGAAACCATCTTCCCAAACCCAGCCGAC 2100
AY713484.1_r2   AGTTCCACGGATGCGTGGATATAGAAACCATCTTCCCAAACCCAGCCGAC 2100
AY198374.1_s    AGTTCCACGGATGCGTGGATATAGAAACCATCTTCCCAAACCCAGCCGAC 2100
AY713483.1_r1   AGTTCCACGGATGCGTGGATATAGAAACCATCTTCCCAAACCCAGCCGAC 2100
AY713485.1_r3   AGTTCCACGGATGCGTGGATATAGAAACCATCTTCCCAAACCCAGCCGAC 2100
                **************************************************

PgCad1_r4       ACCAGAGAGGCCGTGGGGCGAGTGGTAGCGAAGGAGATCCGCCATAACGT 2150
AY713484.1_r2   ACCAGAGAGGCTGTGGGGCGAGTGGTAGCGAAGGAGATCCGCCATAACGT 2150
AY198374.1_s    ACCAGAGAGGCTGTGGGGCGAGTGGTAGCGAAGGAGATCCGCCATAACGT 2150
AY713483.1_r1   ACCAGAGAGGCTGTGGGGCGAGTGGTAGCGAAGGAGATCCGCCATAACGT 2150
AY713485.1_r3   ACCAGAGAGGCTGTGGGGCGAGTGGTAGCGAAGGAGATCCGCCATAACGT 2150
                *********** **************************************

PgCad1_r4       GACCATCGATTTTGAAGAGTTTGAATTTCTCTACCTCACAGTGAGAGTTC 2200
AY713484.1_r2   GACCATCGATTTTGAAGAGTTTGAATTTCTCTACCTCACAGTGAGAGTTC 2200
AY198374.1_s    GACCATCGATTTTGAAGAGTTTGAATTTCTCTACCTCACAGTGAGAGTTC 2200
AY713483.1_r1   GACCATCGATTTTGAAGAGTTTGAATTTCTCTACCTCACAGTGAGAGTTC 2200
AY713485.1_r3   GACCATCGATTTTGAAGAGTTTGAATTTCTCTACCTCACAGTGAGAGTTC 2200
                **************************************************

PgCad1_r4       GAGACTTGCACACAGATGACGGACGAGATTATGATGAATCTACCTTCACG 2250
AY713484.1_r2   GGGACTTGCACACAGATGACGGACGAGATTATGATGAATCTACCTTCACG 2250
AY198374.1_s    GGGACTTGCACACAGATGACGGACGAGATTATGATGAATCTACCTTCACG 2250
AY713483.1_r1   GGGACTTGCACACAGATGACGGACGAGATTATGATGAATCTACCTTCACG 2250
AY713485.1_r3   GGGACTTGCACACAGATGACGGACGAGATTATGATGAATCTACCTTCACG 2250
                * ************************************************

PgCad1_r4       ATAATAATAATAGATATGAACGACAACTGGCCTATCTGGGCGTCTGGTTT 2300
AY713484.1_r2   ATAATAATAATAGATATGAACGACAACTGGCCTATCTGGGCGTCTGGTTT 2300
AY198374.1_s    ATAATAATAATAGATATGAACGACAACTGGCCTATCTGGGCGTCTGGTTT 2300
AY713483.1_r1   ATAATAATAATAGATATGAACGACAACTGGCCTATCTGGGCGTCTGGTTT 2300
AY713485.1_r3   ATAATAATAATAGATATGAACGACAACTGGCCTATCTGGGCGTCTGGTTT 2300
                **************************************************

PgCad1_r4       CCTGAACCAGACCTTCAGTATCCGGGAGCGATCATCTACCGGCGTCGTCA 2350
AY713484.1_r2   CCTGAACCAGACCTTCAGTATCCGGGAGCGATCATCTACCGGCGTCGTCA 2350
AY198374.1_s    CCTGAACCAGACCTTCAGTATTCGGGAGCGATCATCTACCGGCGTCGTCA 2350
AY713483.1_r1   CCTGAACCAGACCTTCAGTATTCGGGAGCGATCATCTACCGGCGTCGTCA 2350
AY713485.1_r3   CCTGAACCAGACCTTCAGTATTCGGGAGCGATCATCTACCGGCGTCGTCA 2350
                ********************* ****************************

PgCad1_r4       TCGGGTCCGTACTCGCTACAGACATTGATGGCCCACTTTACAACCAAGTC 2400
AY713484.1_r2   TCGGGTCCGTACTCGCTACAGACATTGATGGCCCACTTTACAACCAAGTC 2400
AY198374.1_s    TCGGGTCCGTACTCGCTACAGACATTGATGGCCCACTTTACAACCAAGTC 2400
AY713483.1_r1   TCGGGTCCGTACTCGCTACAGACATTGATGGCCCACTTTACAACCAAGTC 2400
AY713485.1_r3   TCGGGTCCGTACTCGCTACAGACATTGATGGCCCACTTTACAACCAAGTC 2400
                **************************************************

PgCad1_r4       CGGTACACCATTATCCCCCAGGAAGATACTCCTGAAGGTCTAGTCCAGAT 2450
AY713484.1_r2   CGGTACACAATTAT------------------------------------ 2414
AY198374.1_s    CGGTACACCATTATCCCCCAGGAAGATACTCCTGAAGGTCTAGTCCAGAT 2450
AY713483.1_r1   CGGTACACCATTATCCCCCAGGAAGATACTCCTGAAGGTCTAGTCCAGAT 2450
AY713485.1_r3   CGGTACACCATTATCCCCCAGGAAGATACTCCTGAAGGTCTAGTCCAGAT 2450
                ******** *****
```

```
PgCad1_r4      ACACTTCGTTACGGGTCAGATTACAGTTGATGAGAATGGTGCAATCGACG 2500
AY713484.1_r2  --------------------------------------------------
AY198374.1_s   ACACTTCGTTACGGGTCAGATTACAGTTGATGAGAATGGTGCAATCGACG 2500
AY713483.1_r1  ACATTTCGTTACGGGTCAGATTACAGTTGATGAGAATGGTGCAATCGACG 2500
AY713485.1_r3  ACACTTCGTTACGGGTCAGATTACAGTTGATGAGAATGGTGCAATCGACG 2500


PgCad1_r4      CTGATATTCCACCTCGTTGGCACCTCAACTACACAGTTATAGCCAGCGAC 2550
AY713484.1_r2  --------------------------------------------------
AY198374.1_s   CTGATATTCCACCTCGTTGGCACCTCAACTACACGGTTATAGCCAGCGAC 2550
AY713483.1_r1  CTGATATTCCACCTCGTTGGCACCTCAACTACACGGTTATAGCCAGCGAC 2550
AY713485.1_r3  CTGATATTCCACCTCGTTGGCACCTCAACTACACGGTTATAGCCAGCGAC 2550


PgCad1_r4      AAATGTTCCGAAGAAAATGAAGAGAACTGTCCCCCGGATCCAGTGTTCTG 2600
AY713484.1_r2  --------------------------------------------------
AY198374.1_s   AAATGTTCCGAAGAAAATGAAGAGAACTGTCCCCCGGATCCAGTGTTCTG 2600
AY713483.1_r1  AAATGTTCTGAAGAAAATGAAGAGAACTGTCCCCCGGATCCAGTGTTCTG 2600
AY713485.1_r3  AAATGTTCCGAAGAAAATGAAGAGAACTGTCCCCCGGATCCAGTGTTCTG 2600


PgCad1_r4      GGATACTCTGGGCGACAACGTAATTAACATCGTGGACATAAACAACAAGG 2650
AY713484.1_r2  ----------------AATGTAATTAACATCGTGGACATAAACAACAAGG 2448
AY198374.1_s   GGATACTCTGGGCGACAATGTAATTAACATCGTGGACATAAACAACAAGG 2650
AY713483.1_r1  GGATACTCTGCGCGACAATGTAATTAACATCGTGGACATAAACAACAAGG 2650
AY713485.1_r3  GGATACTCTGGGCGACAATGTAATTAACATCGTGGACATAAACAACAAGG 2650
                               ** *******************************

PgCad1_r4      TCCCGGCAGCAGACCTCAGTCGATTCAACGAAACGGTGTACATTTATGAA 2700
AY713484.1_r2  TCCCGGCAGCAGACCTCAGTAGATTCAACGAAACGGTGTACATTTATGAA 2498
AY198374.1_s   TCCCGGCAGCAGACCTCAGTCGATTCAACGAAACGGTGTACATTTATGAA 2700
AY713483.1_r1  TCCCGGCAGCAGACCTCAGTCGATTCAACGAAACGGTGTACATTTATGAA 2700
AY713485.1_r3  TCCCGGCAGCAGACCTCAGTCGATTCAACGAAACGGTGTACATTTATGAA 2700
               ******************** *****************************

PgCad1_r4      AATGCACCCGATTTCACAAACGTGGTCAAGATATACTCCATCGACGAAGA 2750
AY713484.1_r2  AATGCACCCGATTTCACAAACGTGGTCAAGATATACTCCATCGACGAAGA 2548
AY198374.1_s   AATGCACCCGATTTCACAAACGTGGTCAAGATATACTCCATCGACGAAGA 2750
AY713483.1_r1  AATGCACCCGATTTCACAAACGTGGTCAAGATATACTCCATCGACGAAGA 2750
AY713485.1_r3  AATGCACCCGATTTCACAAACGTGGTCAAGATATACTCCATCGACGAAGA 2750
               **************************************************

PgCad1_r4      CAGAGACGAAATATATCACACGGTGCGGTACCAGATCAATTATGCTGTGA 2800
AY713484.1_r2  CAGAGACGAAATATATCACACGGTGCGGTACCAGATCAATTATGCTGTGA 2598
AY198374.1_s   CAGAGACGAAATATATCACACGGTGCGGTACCAGATCAATTATGCTGTGA 2800
AY713483.1_r1  CAGAGACGAAATATATCACACGGTGCGGTACCAGATCAATTATGCTGTGA 2800
AY713485.1_r3  CAGAGACGAAATATATCACACGGTGCGGTACCAGATCAATTATGCTGTGA 2800
               **************************************************

PgCad1_r4      ACCAGCGGCTGCGAGACTTCTTCGCC---------------GGTGAGGTG 2835
AY713484.1_r2  ACCAGCGGCTGCGAGACTTCTTCGCCATAGACCTGGATTCAGGCCAGGTG 2648
AY198374.1_s   ACCAGCGGCTGCGAGACTTCTTCGCCATAGACCTGGATTCAGGCCAGGTG 2850
AY713483.1_r1  ACCAGCGGCTGCGAGACTTCTTCGCCATAGACCTGGATTCAGGCCAGGTG 2850
AY713485.1_r3  ACCAGCGGCTGCGAGACTTCTTCGCCATAGACCTGGATTCAGGCCAGGTG 2850
               **************************               **  *****

PgCad1_r4      TACGTGGAGAACACCAACAATGAGCTCCTGGATCGGGACAGAGGCGAAGA 2885
AY713484.1_r2  TACGTGGAGAACACCAACAATGAGCTCCTGGATCGGGACAGAGGCGAAGA 2698
AY198374.1_s   TACGTGGAGAACACCAACAATGAGCTCCTGGATCGGGACAGAGGCGAAGA 2900
AY713483.1_r1  TACGTGGAGAACACCAACAATGAGCTCCTGGATCGGGACAGAGGCGAAGA 2900
AY713485.1_r3  TACGTGGAGAACACCAACAATGAGCTCCTGGATCGGGACAGAGGCGAAGA 2900
               **************************************************

PgCad1_r4      CCAACACAGGATATTCATTAACCTCATTGACAACTTTTATAGAGAAGGAG 2935
AY713484.1_r2  CCAACACAGGATATTCATTAACCTCATTGACAACTTTTATAGCGAAGGAG 2748
AY198374.1_s   CCAACACAGGATATTCATTAACCTCATTGACAACTTTTATAGCGAAGGAG 2950
AY713483.1_r1  CCAACACAGGATATTCATTAACCTCATTGACAACTTTTATAGCGAAGGAG 2950
AY713485.1_r3  CCAACACAGGATATTCATTAACCTCATTGACAACTTTTATAGCGAAGGAG 2950
               ****************************************** *******
```

```
PgCad1_r4       ATGGAAATAGAAATGTAAACACTACAGAGGTGCTGGTGATACTATTAGAT 2985
AY713484.1_r2   ATGGAAATAGAAATGTAAACACTACAGAGGTGCTGGTGATACTATTAGAT 2798
AY198374.1_s    ATGGAAATAGAAATGTAAACACTACAGAGGTGCTGGTGATACTATTAGAT 3000
AY713483.1_r1   ATGGAAATAGAAATGTAAACACTACAGAGGTGCTGGTGATACTATTAGAT 3000
AY713485.1_r3   ATGGAAATAGAAATGTAAACACTACAGAGGTGCTGGTGATACTATTAGAT 3000
                **************************************************

PgCad1_r4       GAGAATGACAACGCTCCTGAATTGCCGACTCCAGAAGAGCTGAGTTGGAG 3035
AY713484.1_r2   GAGAATGACAACGCTCCTGAATTGCCGACTCCAGAAGAGCTGAGTTGGAG 2848
AY198374.1_s    GAGAATGACAACGCTCCTGAATTGCCGACTCCAGAAGAGCTGAGTTGGAG 3050
AY713483.1_r1   GAGAATGACAACGCTCCTGAATTGCCGACTCCAGAAGAGCTGAGTTGGAG 3050
AY713485.1_r3   GAGAATGACAACGCTCCTGAATTGCCGACTCCAGAAGAGCTGAGTTGGAG 3050
                **************************************************

PgCad1_r4       CATTTCCGAGAATTTACAAGAGGGTATAACACTCGATGGCGAAAGCGATG 3085
AY713484.1_r2   CATTTCCGAGAATTTACAAGAGGGTATAACACTCGATGGCGAAAGCGATG 2898
AY198374.1_s    CATTTCCGAGAATTTACAAGAGGGTATAACACTCGATGGCGAAAGCGATG 3100
AY713483.1_r1   CATTTCCGAGAATTTACAAGAGGGTATAACACTCGATGGCGAAAGCGATG 3100
AY713485.1_r3   CATTTCCGAGAATTTACAAGAGGGTATAACACTCGATGGCGAAAGCGATG 3100
                **************************************************

PgCad1_r4       TGATATACGCACCGGATATAGACGAAGAGGACACGCCAAACTCTCACGTT 3135
AY713484.1_r2   TGATATACGCACCGGATATAGACGAAGAGGACACGCCAAACTCTCACGTT 2948
AY198374.1_s    TGATATACGCACCGGATATAGACGAAGAGGACACGCCAAACTCTCACGTT 3150
AY713483.1_r1   TGATATACGCACCGGATATAGACGAAGAGGACACGCCAAACTCTCACGTT 3150
AY713485.1_r3   TGATATACGCACCGGATATAGACGAAGAGGACACGCCAAACTCTCACGTT 3150
                **************************************************

PgCad1_r4       GGCTACGCAATCCTGGCCATGACAGTCACCAATAGAGACCTGGACACTGT 3185
AY713484.1_r2   GGCTACGCAATCCTGGCCATGACAGTCACCAATAGAGACCTGGACACTGT 2998
AY198374.1_s    GGCTACGCAATCCTGGCCATGACAGTCACCAATAGAGACCTGGACACTGT 3200
AY713483.1_r1   GGCTACGCAATCCTGGCCATGACAGTCACCAATAGAGACCTGGACACTGT 3200
AY713485.1_r3   GGCTACGCAATCCTGGCCATGACAGTCACCAATAGAGACCTGGACACTGT 3200
                **************************************************

PgCad1_r4       TCCGAGACTTCTCAACATGCTGTCGCCTAACAACGTAACCGGATTCCTTC 3235
AY713484.1_r2   TCCGAGACTTCTCAACATGCTGTCGCCTAACAACGTAACCGGATTCCTTC 3048
AY198374.1_s    TCCGAGACTTCTCAACATGCTGTCGCCTAACAACGTAACCGGATTCCTCC 3250
AY713483.1_r1   TCCGAGACTTCTCAACATGCTGTCGCCTAACAACGTAACCGGATTCCTCC 3250
AY713485.1_r3   TCCGAGACTTCTCAACATGCTGTCGCCTAACAACGTAACCGGATTCCTCC 3250
                ************************************************ *

PgCad1_r4       AGACAGCAATGCCTTTGAGAGGATATTGGGGTACTTACGATATAAGTATA 3285
AY713484.1_r2   AGACAGCAATGCCTTTGAGAGGATATTGGGGTACTTACGATATAAGTATA 3098
AY198374.1_s    AGACAGCAATGCCTTTGAGAGGATATTGGGGGACTTACGATATAAGTATA 3300
AY713483.1_r1   AGACAGCAATGCCTTTGAGAGGATATTGGGGGACTTACGATATAAGTGTA 3300
AY713485.1_r3   AGACAGCAATGCCTTTGAGAGGATATTGGGGGACTTACGATATAAGTGTA 3300
                ******************************* *************** **

PgCad1_r4       CTGGCGTTCGACCACGGTATTCCTCAGCAGATATCTCATGAGGTGTATGA 3335
AY713484.1_r2   CTGGCGTTCGACCACGGTATTCCTCAGCAGATATCTCATGAGGTGTATGA 3148
AY198374.1_s    CTGGCGTTCGACCACGGTATTCCTCAGCAGATATCTCATGAGGTGTATGA 3350
AY713483.1_r1   CTGGCGTTCGACCACGGTATTCCTCAGCAGATATCTCATGAGGTGTATGA 3350
AY713485.1_r3   CTGG---------------------------------------------- 3304
                ****

PgCad1_r4       ACTGGAAATTCGACCTTACAATTACAATCCTCCCCAGTTCGTTTTTCCTG 3385
AY713484.1_r2   ATTGGAAATTCGACCTTACAATTACAATCCTCCCCAGTTCGTTTTTCCTG 3198
AY198374.1_s    ATTGGAAATTCGACCTTACAATTACAATCCTCCCCAGTTCGTTTTTCCTG 3400
AY713483.1_r1   ATTGGAAATTCGACCTTACAATTACAATCCTCCCC--------------- 3385
AY713485.1_r3   --------------------------------------------------

PgCad1_r4       AATCCGGGACGATTCTACGACTGGCTTTGGAACGCGCAGTGGTAAATAAT 3435
AY713484.1_r2   AATCCGGGACGATTCTACGACTGGCTTTGGAACGCGCAGTGGTAAATAAT 3248
AY198374.1_s    AATCCGGGACGATTCTACGACTGGCTTTGGAACGCGCAGTGGTAAATAAT 3450
AY713483.1_r1   -------GACGA--CTACGACTGGCTTTGGAACGCGCTGTGGTAAATAAT 3426
AY713485.1_r3   ------------------------------AACGCGCAGTGGTAAATAAT 3324
                                              ******* ************
```

```
PgCad1_r4       GTTTTGTCACTTGTAAACGGTGACCCGTTAGACAGGATACAAGCAATTGA 3485
AY713484.1_r2   GTTTTGTCACTTGTAAACGGTGACCCGTTAGACAGGATACAAGCAATTGA 3298
AY198374.1_s    GTTTTGTCACTTGTAAACGGTGACCCGTTAGACAGGATACAAGCAATTGA 3500
AY713483.1_r1   GTATTGTCACTTGTAAACGGTGACCTGTTAGACAGGATACAAGCAATTGA 3476
AY713485.1_r3   GTTTTGTCACTTGTAAACGGTGACCCGTTAGACAGGATACAAGCAATTGA 3374
                ** ********************** ************************

PgCad1_r4       CGACGATGGTCTTGATGCTGGCGTGGTGACTTTCGATATTGTTGGAGATG 3535
AY713484.1_r2   CGACGATGGTCTTGATGCTGGCGTGGTGACTTTCGATATTGTTGGAGATG 3348
AY198374.1_s    CGACGATGGTCTTGATGCTGGCGTGGTGACTTTCGATATTGTTGGAGATG 3550
AY713483.1_r1   CGACGATGGTCTCGATGCTGGCGTGGTGACTTTCGATATTGTTGGAGATG 3526
AY713485.1_r3   CGACGATGGTCTTGATGCTGGCGTGGTGACTTTCGATATTGTTGGAGATG 3424
                ************ *************************************

PgCad1_r4       CTGATGCATCAAGCTACTTCAGAGTAAATAATGATGGCGACAACTTTGGG 3585
AY713484.1_r2   CTGATGCATCAAACTACTTCAGAGTAAATAATGATGGCGACAACTTTGGA 3398
AY198374.1_s    CTGATGCATCAAACTACTTCAGAGTAAATAATGATGGCGACAACTTTGGA 3600
AY713483.1_r1   CTGATGCATCAAACTACTTCAGAGTAAATAATGATGGCGACAACTTTGGA 3576
AY713485.1_r3   CTGATGCATCAAACTACTTCAGAGTAAATAATGATGGCGACAACTTTGGA 3474
                ************ ************************************ 

PgCad1_r4       ACCTTGTTGCTGACACAGGCGCTTCCTGAGGAAGGCAAGGAATTTGAGGT 3635
AY713484.1_r2   ACCTTGTTGCTGACACAGGCGCTTCCTGAGGAAGGCAAGGAATTTGAGGT 3448
AY198374.1_s    ACCTTGTTGCTGACACAGGCGCTTCCTGAGGAAGGCAAGGAATTTGAGGT 3650
AY713483.1_r1   ACCTTGTTGCTGACACAGGCGCTTCCTGAGGAAGGCAAGGAATTTGAGGT 3626
AY713485.1_r3   ACCTTGTTGCTGACACAGGCGCTTCCTGAGGAAGGCAAGGAATTTGAGGT 3524
                **************************************************

PgCad1_r4       TACCATCCGGGCTACAGACGGCGGAACGGAACCTCGATCATATTCAACAG 3685
AY713484.1_r2   TACCATCCGGGCTACAGACGGCGGAACAGAACCTCGATCATATTCAACAG 3498
AY198374.1_s    TACCATCCGGGCTACAGACGGCGGAACAGAACCTCGATCATATTCAACAG 3700
AY713483.1_r1   TACCATCCGGGCTACAGACGGCGGAACAGAACCTCGATCATATTCAACAG 3676
AY713485.1_r3   TACCATCCGGGCTACAGACGGCGGAACAGAACCTCGATCATATTCAACAG 3574
                *************************** **********************

PgCad1_r4       ACTCCACTATAACAGTGCTCTTCGTTCCGACTTTGGGTGATCCGATCTTT 3735
AY713484.1_r2   ACTCCACTATAACAGTGCTCTTCGTTCCGACTTTGGGTGATCCGATCTTT 3548
AY198374.1_s    ACTCCACTATAACAGTGCTCTTCGTTCCGACTTTGGGTGATCCGATCTTT 3750
AY713483.1_r1   ACTCCACTATAACAGTCCTCTTCGTTCCGACTTTGGGTGATCCGATCTTT 3726
AY713485.1_r3   ACTCCACTATAACAGTGCTCTTCGTTCCGACTTTGGGTGATCCGATCTTT 3624
                **************** *********************************

PgCad1_r4       CAAGATAACACTTACTCAGTAGCATTCTTTGAAAAAGAGGTTGGCTTGAC 3785
AY713484.1_r2   CAAGATAACACTTACTCAGTAGCATTCTTTGAAAAAGAGGTTGGCTTGAC 3598
AY198374.1_s    CAAGATAACACTTACTCAGTAGCATTCTTTGAAAAAGAGGTTGGCTTGAC 3800
AY713483.1_r1   CAAGATAACACTTACTCAGTAGCATTCTTTGAAAAAGAGGTTGGCTTGAC 3776
AY713485.1_r3   CAAGATAACACTTACTCAGTAGCATTCTTTGAAAAAGAGGTTGGCTTGAC 3674
                **************************************************

PgCad1_r4       TGAGAGGTTCTCGCTCCCACATGCAGAGGACCCTAAGAACAAACTCTGCA 3835
AY713484.1_r2   TGAGAGGTTCTCGCTCCCACATGCAGAGGACCCTAAGAACAAACTCTGCA 3648
AY198374.1_s    TGAGAGGTTCTCGCTCCCACATGCAGAGGACCCTAAGAACAAACTCTGCA 3850
AY713483.1_r1   TGAGAGGTTCTCGCTCCCACATGCAGAGGACCCTAAGAACAAACTCTGCA 3826
AY713485.1_r3   TGAGAGGTTCTCGCTCCCACATGCAGAGGACCCTAAGAACAAACTCTGCA 3724
                **************************************************

PgCad1_r4       CTGACGACTGTCACGATATTTACTACAGGATCTTTGGTGGTGTGGATTAC 3885
AY713484.1_r2   CTGACGACTGTCACGATATTTACTACAGGATCTTTGGTGGTGTGGATTAC 3698
AY198374.1_s    CTGACGACTGTCACGATATTTACTACAGGATCTTTGGTGGTGTGGATTAC 3900
AY713483.1_r1   CTGACGACTGTCACGATATTTACTACAGGATCTTTGGTGGTGTGGATTAC 3876
AY713485.1_r3   CTGACGACTGTCACGATATTTACTACAGGATCTTTGGTGGTGTGGATTAC 3774
                **************************************************

PgCad1_r4       GAGCCATTTGACCTGGACCCGGTGACGAACGTGATCTTCCTGAAATCAGA 3935
AY713484.1_r2   GAGCCATTTGACCTGGACCCGGTGACGAACGTGATCTTCCTGAAATCAGA 3748
AY198374.1_s    GAGCCATTTGACCTGGACCCGGTGACGAACGTGATCTTCCTGAAATCAGA 3950
AY713483.1_r1   GAGCCATTTGACCTGGACCCGGTGACGAACGTGATCTTCCTGAAATCAGA 3926
AY713485.1_r3   GAGCCATTTGACCTGGACCCGGTGACGAACGTGATCTTCCTGAAATCAGA 3824
                **************************************************
```

```
PgCad1_r4       ACTAGACCGGGAGACCACTGCCACGCATGTGGTGCAAGTGGCAGCCAGTA 3985
AY713484.1_r2   ACTAGACCGGGAGACCACTGCTACGCATGTGGTGCAAGTGGCAGCCAGTA 3798
AY198374.1_s    ACTAGACCGGGAGACCACTGCTACGCATGTGGTGCAAGTGGCAGCCAGTA 4000
AY713483.1_r1   ACTGGACCGAGAGACCACTGCCACGCATGTGGTTCAAGTGGCTGCCAGTA 3976
AY713485.1_r3   ACTAGACCGGGAGACCACTGCTACGCATGTGGTGCAAGTGGCAGCCAGTA 3874
                *** ***** *********** *********** ******** *******

PgCad1_r4       ATTCGCCCACAGGAGGCGGAATACCACTCCCTGGGTCTCTTCTCACCGTC 4035
AY713484.1_r2   ATTCGCCCACAGGAGGCGGAATACCACTCCCTGGGTCTCTTCTCACCGTC 3848
AY198374.1_s    ATTCGCCCACAGGAGGCGGAATACCACTCCCTGGGTCTCTTCTCACCGTC 4050
AY713483.1_r1   ATTCGCCCACAGGAGGCGGAATACCACTCCCTGGGTCTCTTCTCACCGTC 4026
AY713485.1_r3   ATTCGCCCACAGGAGGCGGAATACCACTCCCTGGGTCTCTTCTCACCGTC 3924
                **************************************************

PgCad1_r4       ACTGTCACTGTACGAGAAGCGGATCCACGGCCTGTGTTCGAGCAGCGTCT 4085
AY713484.1_r2   ACTGTCACTGTACGAGAAGCGGATCCACGGCCTGTGTTCGAGCAGCGTCT 3898
AY198374.1_s    ACTGTCACTGTACGAGAAGCGGATCCACGGCCTGTGTTCGAGCAGCGTCT 4100
AY713483.1_r1   ACTGTCACTGTACGAGAAGCGGATCCACGGCCTGTGTTCGAGCAGCGTCT 4076
AY713485.1_r3   ACTGTCACTGTACGAGAAGCGGATCCACGGCCTGTGTTCGAGCAGCGTCT 3974
                **************************************************

PgCad1_r4       GTACACGGCTGGCATTTCCACTTCCGATAACATCAACAGGGAACTACTCA 4135
AY713484.1_r2   GTACACGGCTGGCATTTCCACTTCCGATAACATCAACAGGGAACTACTCA 3948
AY198374.1_s    GTACACGGCTGGCATTTCCACTTCCGATAACATCAACAGGGAACTACTCA 4150
AY713483.1_r1   GTACACGGCTGGCATTTCCACTTCCGATAACATCAACAGAGAACTACTCA 4126
AY713485.1_r3   GTACACGGCTGGCATTTCCACTTCCGATAACATCAACAGAGAACTACTCA 4024
                *************************************** **********

PgCad1_r4       CCGTTCGTGCAACTCATTCCGAAAACGCACAATTGACATATACCATCGAA 4185
AY713484.1_r2   CCGTTCGTGCAACTCATTCCGAAAACGCACAATTGACATATACCATCGAA 3998
AY198374.1_s    CCGTTCGTGCAACTCATTCCGAAAACGCACAATTGACATATACCATCGAA 4200
AY713483.1_r1   CCGTTCGTGCAACTCATTCCGAAAACGCACAATTGACATATACCATCGAA 4176
AY713485.1_r3   CCGTTCGTGCAACTCATTCCGAAAACGCACAATTGACATATACCATCGAA 4074
                **************************************************

PgCad1_r4       GACGGTTCTATGGCGGTGGACTCCACTCTGGAAGCCGTCAAGGACTCGGC 4235
AY713484.1_r2   GACGGTTCTATGGCGGTGGACTCCACTCTGGAAGCCGTCAAGGACTCGGC 4048
AY198374.1_s    GACGGTTCTATGGCGGTGGACTCCACTCTGGAAGCCGTCAAGGACTCGGC 4250
AY713483.1_r1   GATGGTTCTATGGTGGTGGACTCCACTCTGGAAGCCGTCAAGGACTCGGC 4226
AY713485.1_r3   GACGGTTCTATGGCGGTGGACTCCACTCTGGAAGCCGTCAAGGACTCGGC 4124
                ** ********** ************************************

PgCad1_r4       GTTCCATCTGAACGCGCAGACCGGCGTCCTCATACTGAGGATACAACCTA 4285
AY713484.1_r2   GTTCCATCTGAACGCGCAGACCGGCGTCCTCATACTGAGGATACAACCTA 4098
AY198374.1_s    GTTCCATCTGAACGCGCAGACCGGCGTCCTCATACTGAGGATACAACCTA 4300
AY713483.1_r1   GTTCCATCTGAACGCGCAGACCGGCGTCCTCATACTGAGGATACAACCTA 4276
AY713485.1_r3   GTTCCATCTGAACGCGCAGACCGGCGTCCTCATACTGAGGATACAACCTA 4174
                **************************************************

PgCad1_r4       CTGCCAGCATGCAGGGCATGTTCGAGTTCAACGTCATCGCTACTGATCCA 4335
AY713484.1_r2   CTGCCAGCATGCAGGGCATGTTCGAGTTCAACGTCATCGCTACTGATCCA 4148
AY198374.1_s    CTGCCAGCATGCAGGGCATGTTCGAGTTCAACGTCATCGCTACTGATCCA 4350
AY713483.1_r1   CTGCCAGCATGCAGGGCATGTTTGAGTTCAACGTCATCGCTACTGACCCA 4326
AY713485.1_r3   CTGCCAGCATGCAGGGCATGTTCGAGTTCAACGTCATCGCTACTGATCCA 4224
                ********************** *********************** ***

PgCad1_r4       GATGAGAAGACAGATACGGCAGAGGTGAAAGTCTACCTCATTTCATCCCA 4385
AY713484.1_r2   GATGAGAAGACAGATACGGCAGAGGTGAAAGTCTACCTCATTTCATCCCA 4198
AY198374.1_s    GATGAGAAGACAGATACGGCAGAGGTGAAAGTCTACCTCATTTCATCCCA 4400
AY713483.1_r1   GATGAGAAGACAGATACGGCAGAGGTGAAAGTCTACCTCATTTCATCCCA 4376
AY713485.1_r3   GATGAGAAGACAGATACGGCAGAGGTGAAAGTCTACCTCATTTCATCCCA 4274
                **************************************************

PgCad1_r4       AAATAGGGTGTCCTTCATATTCCTGAACGATGTGGAGACGGTTGAGAGTA 4435
AY713484.1_r2   AAATAGGGTGTCCTTCATATTCCTGAACGATGTGGAGACGGTTGAGAGTA 4248
AY198374.1_s    AAATAGGGTGTCCTTCATATTCCTGAACGATGTGGAGACGGTTGAGAGTA 4450
AY713483.1_r1   AAATAGGGTGTCCTTCATATTCCTGAACGATGTGGAGACTGTTGAGAGTA 4426
AY713485.1_r3   AAATAGGGTGTCCTTCATATTCCTGAACGATGTGGAGACGGTTGAGAGTA 4324
                *************************************** **********
```

```
PgCad1_r4       ACAGAGACTTTATCGCAGAAACGTTCAGCGTTGGCTTCAACATGACCTGC 4485
AY713484.1_r2   ACAGAGACTTTATCGCAGAAACGTTCAGCGTTGGCTTCAACATGACCTGC 4298
AY198374.1_s    ACAGAGACTTTATCGCAGAAACGTTCAGCGTTGGCTTCAACATGACCTGC 4500
AY713483.1_r1   ACAGAGACTTTATCGCAGAAACGTTCAGCGTTGGCTTCAACATGACCTGC 4476
AY713485.1_r3   ACAGAGACTTTATCGCAGAAACGTTCAGCGTTGGCTTCAACATGACCTGC 4374
                **************************************************

PgCad1_r4       AATATAGATCAGGTGCTGCCGGGCACCAACGGCGCCGGGGTGATTCAGGA 4535
AY713484.1_r2   AATATAGATCAGGTGCTGCCGGGCACCAACGACGCCGGGGTGATTCAGGA 4348
AY198374.1_s    AATATAGATCAGGTGCTGCCGGGCACCAACGACGCCGGGGTGATTCAGGA 4550
AY713483.1_r1   AATATAGATCAGGTGCTGCCGGGCACCAACGACGCCGGGGTGATTCAGGA 4526
AY713485.1_r3   AATATAGATCAGGTGCTGCCGGGCACCAACGACGCCGGGGTGATTCAGGA 4424
                ******************************* ******************

PgCad1_r4       GGCCATGGCGGAAGTCCATGCTCACTTCATACAGGATAACATCCCTGTGA 4585
AY713484.1_r2   GGCCATGGCGGAAGTCCATGCTCACTTCATACAGGATAACATCCCTGTGA 4398
AY198374.1_s    GGCCATGGCGGAAGTCCATGCTCACTTCATACAGGATAACATCCCTGTGA 4600
AY713483.1_r1   GGCCATGGCGGAAGTCCATGCTCACTTCATACAGGATAACATCCCTGTGA 4576
AY713485.1_r3   GGCCATGGCGGAAGTCCATGCTCACTTCATACAGGATAACATCCCTGTGA 4474
                **************************************************

PgCad1_r4       GCGCCGACAGTATTGAAGAGCTTCGCAGTGACACTCAGCTGCTGCGCTCC 4635
AY713484.1_r2   GCGCCGACAGTATTGAAGAGCTTCGCAGTGACACTCAGCTGCTGCGCTCC 4448
AY198374.1_s    GCGCCGACAGTATTGAAGAGCTTCGCAGTGACACTCAGCTGCTGCGCTCC 4650
AY713483.1_r1   GCGCCGACAGTATTGAAGAGCTTCGCAGTGACACTCAGCTGCTGCGCTCC 4626
AY713485.1_r3   GCGCCGACAGTATTGAAGAGCTTCGCAGTGACACTCAGCTGCTGCGCTCC 4524
                **************************************************

PgCad1_r4       GTCCAAGGTGTGTTGAACCAGCGGCTGTTGGTCCTGAACGACCTGGTGAC 4685
AY713484.1_r2   GTCCAAGGTGTGTTGAACCAACGGCTGTTGGTCCTGAACGACCTGGTGAC 4498
AY198374.1_s    GTCCAAGGTGTGTTGAACCAACGGCTGTTGGTCCTGAACGACCTGGTGAC 4700
AY713483.1_r1   GTCCAAGGTGTGTTGAACCAACGGCTGTTGGTCCTGAACGACCTGGTGAC 4676
AY713485.1_r3   GTCCATGGTGTGTTGAACCAACGGCTGTTGGTCCTGAACGACCTGGTGAC 4574
                ***** ************** *****************************

PgCad1_r4       GGGGGTCAGCCCTGATCTCGGCACTGCCGGCGTGCAGATCACCATCTATG 4735
AY713484.1_r2   GGGGGTCAGCCCTGATCTCGGCACTGCCGGCGTGCAGATCACCATCTATG 4548
AY198374.1_s    GGGGGTCAGCCCTGATCTCGGCACTGCCGGCGTGCAGATCACCATCTATG 4750
AY713483.1_r1   CGGGGTCAGCCCTGATCTCGGCACTGCCGGCGTGCAGATCACCATCTATG 4726
AY713485.1_r3   GGGGGTCAGCCCTGATCTCGGCACTGCCGGCGTGCAGATCACCATCTATG 4624
                 *************************************************

PgCad1_r4       TGCTAGCCGGGTTGTCAGCCATCCTTGCCTTCCTGTGCCTTATTCTGCTC 4785
AY713484.1_r2   TGCTAGCCGGGTTGTCAGCCATCCTTGCCTTCCTGTGCCTTATTCTGCTC 4598
AY198374.1_s    TGCTAGCCGGGTTGTCAGCCATCCTTGCCTTCCTGTGCCTTATTCTGCTC 4800
AY713483.1_r1   TGCTAGCCGGGTTGTCAGCCATCCTTGCCTTCCTGTGCCTTATTCTGCTC 4776
AY713485.1_r3   TGCTAGCCGGGTTGTCAGCCATCCTTGCCTTCCTGTGCCTTATTCTGCTC 4674
                **************************************************

PgCad1_r4       ATCACATTCATCGTGAGGACCCGAGCTCTGAACCGCCGTTTGGAAGCACT 4835
AY713484.1_r2   ATCACATTCATCGTGAGGACCCGAGCTCTGAACCGCCGTTTGGAAGCACT 4648
AY198374.1_s    ATCACATTCATCGTGAGGACCCGAGCTCTGAACCGCCGTTTGGAAGCACT 4850
AY713483.1_r1   ATCACATTCATCGTGAGGACCCGAGCTCTGAACCGCCGTTTGGAAGCACT 4826
AY713485.1_r3   ATCACATTCATCGTGAGGACCCGAGCTCTGAACCGCCGTTTGGAAGCACT 4724
                **************************************************

PgCad1_r4       GTCGATGACGAAATACGGCTCGGTGGATTCGGGGCTGAACCGAGTGGGGA 4885
AY713484.1_r2   GTCGATGACGAAATACGGCTCGGTGGATTCGGGGCTGAACCGAGTGGGGA 4698
AY198374.1_s    GTCGATGACGAAATACGGCTCGGTGGATTCGGGGCTGAACCGAGTGGGGA 4900
AY713483.1_r1   GTCGATGACGAAATACGGCTCGGTGGATTCGGGGCTGAACCGAGTGGGGA 4876
AY713485.1_r3   GTCGATGACGAAATACGGCTCGGTGGATTCGGGGCTGAACCGAGTGGGGA 4774
                **************************************************

PgCad1_r4       TAGCGGCCCCAGGAACCAACAAACACGCCATCGAAGGCTCCAACCCCATC 4935
AY713484.1_r2   TAGCGGCCCCAGGAACCAACAAACACGCCATCGAAGGCTCCAACCCCATC 4748
AY198374.1_s    TAGCGGCCCCAGGAACCAACAAACACGCCATCGAAGGCTCCAACCCCATC 4950
AY713483.1_r1   TAGCGGCCCCAGGAACCAACAAACACGCCATCGAAGGCTCCAACCCCATC 4926
AY713485.1_r3   TAGCGGCCCCAGGAACCAACAAACACGCCATCGAAGGCTCCAACCCCATC 4824
                **************************************************
```

```
PgCad1_r4       TGGAACGAGCAGATCAAGGCCCCGGACTTCGATGCCATCAGTGACACATC 4985
AY713484.1_r2   TGGAACGAGCAGATCAAGGCCCCGGACTTCGATGCCATCAGTGACACATC 4798
AY198374.1_s    TGGAACGAGCAGATCAAGGCCCCGGACTTCGATGCCATCAGTGACACATC 5000
AY713483.1_r1   TGGAACGAGCAGATCAAGGCCCCGGACTTCGATGCCATCAGTGACACATC 4976
AY713485.1_r3   TGGAACGAGCAGATCAAGGCCCCGGACTTCGATGCCATCAGTGACACATC 4874
                **************************************************

PgCad1_r4       TGACGAGTCTGATCTGATCGGCATCGAGGATCTACCACAATTCAAGAGCG 5035
AY713484.1_r2   TGACGAGTCTGATCTGATCGGCATCGAGGATCTACCACAATTCAAGAGCG 4848
AY198374.1_s    TGACGAGTCTGATCTGATCGGCATCGAGGATCTACCACAATTCAAGAGCG 5050
AY713483.1_r1   TGACGAGTCTGATCTGATCGGCATCGAGGATCTACCACAATTCAAGAGCG 5026
AY713485.1_r3   TGACGAGTCTGATCTGATCGGCATCGAGGATCTACCACAATTCAGGAGCG 4924
                ******************************************** *****

PgCad1_r4       ACTATTTCCCGCCTGAGGACTCGGAATCCGCTCACGCCGCCTTTAGCGAC 5085
AY713484.1_r2   ACTATTTCCCGCCTGAGGACTCGGAATCCGCTCACGCCGCCTTTAGCGAC 4898
AY198374.1_s    ACTATTTCCCGCCTGAGGACTCGGAATCCGCTCACGCCGCCTTTAGCGAC 5100
AY713483.1_r1   ACTATTTCCCGCCTGAGGACTCGGAATCCGCTCACGCCGCCTTTAGCGAC 5076
AY713485.1_r3   ACTATTTCCCGCCTGAGGACTCGGAGTCCGCTCACGCCGCCTTTAGCGAC 4974
                ************************* ************************

PgCad1_r4       CGCACGCCACGCGGGAACGATGCGCCTATTGCACACAGTAGCAACAACTT 5135
AY713484.1_r2   CGCACGCCACGCGGGAACGATGCGCCTATTGCACACAGTAGCAACAACTT 4948
AY198374.1_s    CGCACGCCACGCGGGAACGATGCGCCTATTGCACACAGTAGCAACAACTT 5150
AY713483.1_r1   CGCACGCCACGCGGGAACGATGCGCCTATTGCACACAGTAGCAACAACTT 5126
AY713485.1_r3   CGCACGCCACGCGGGAACGATGCGCCTATTGCACACAGTAGCAACAACTT 5024
                **************************************************

PgCad1_r4       CGGTTTCAACACCAGCCCTTTTAGCGCGGAGTTCACTAACAGGCGCATGC 5185
AY713484.1_r2   CGGTTTCAACACCAGTCCTTTTAGCGCGGAGTTCACTAACAGGCGCATGC 4998
AY198374.1_s    CGGTTTCAACACCAGCCCTTTTAGCGCGGAGTTCACTAACAGGCGCATGC 5200
AY713483.1_r1   CGGTTTCAACACCAGCCCTTTTAGCGCGGAGTTCACTAACAGGCGCATGC 5176
AY713485.1_r3   CGGTTTCAACACCAGCCCTTTTAGCGCGGAGTTCACTAACAGGCGCATGC 5074
                *************** **********************************

PgCad1_r4       GACCATAG 5193
AY713484.1_r2   GACCATAG 5006
AY198374.1_s    GACCATAG 5208
AY713483.1_r1   GACCATAG 5184
AY713485.1_r3   GACCATAG 5082
                ********
```
