# Supplementary material for: Similar Genetic Basis of Resistance to Bt Toxin Cry1Ac in Boll-Selected and Diet-Selected Strains of Pink Bollworm
Source: PLoS One. 2012 Apr 18;7(4):e35658. doi: 10.1371/journal.pone.0035658 (PMC3329465; doi:10.1371/journal.pone.0035658)
Supplement: Figure S3 — Sequence alignment of translated PgCad1 cadherin alleles. The CLUSTAL W multiple sequence alignment program was used to align deduced amino acid sequences corresponding to the PgCad1 s (AY198374.1), r1 (AY713483.1), r2 (AY713484.1), r3 (AY713485.1), and r4 cadherin alleles. Residues conserved in all of the sequences are marked with “*” and conservative substitutions are indicated with “:” or “.”. The location of the r4 deletion resulting in loss of 5 residues from PgCad1 is highlighted in red and amino acid the 14 amino acid substitutions that are unique to r4 are highlighted in green. (PDF) [file pone.0035658.s003.pdf]

```
CLUSTAL 2.1 multiple sequence alignment

AY19373.1_R1      MAGDACILVTVLLTFATSVFGQETTSSRCYYMTDAIPREPKPDDLPDLEWTGGWTDWPLI 60
AY19374.1_R2      MAGDACILVTVLLIFAISVFGQETASSRCYYMTDAIPREPKPDDLPDLEWTGGWTDWPLI 60
AY19375.1_R3      MAGDACILVTVLLTFATSVFGQETTSSRCYYMTDAIPREPKPDDLPDLEWTGGWTDWPLI 60
AY198374.1_S      MAGDACILVTVLLTFATSVFGQETTSSRCYYMTDAIPREPKPDDLPDLEWTGGWTDWPLI 60
PgCad1_R4         MAGDACILVTVLLTFATLVFGQETASSRCYYMTDAIPREPKPDDLPDLEWTGGWTDWPLI 60
                  ************* **  ******:***********************************

AY19373.1_R1      PAEPRDDVCINGWYPQLTSTSLGTIIIHMEEEIEGDVAIAKLNYDGSGTPEIVQPMVIGS 120
AY19374.1_R2      PAEPRDDVCINGWYPQLTSTSLGTIIIHMEEEIEGDVAIAKLNYDGSGTPEIVQPMVIGS 120
AY19375.1_R3      PAEPRDDVCINGWYPQLTSTSLGTIIIHMEEEIEGDVAIAKLNYDGSGTPEIVQPMVIGS 120
AY198374.1_S      PAEPRDDVCINGWYPQLTSTSLGTIIIHMEEEIEGDVAIAKLNYDGSGTPEIVQPMVIGS 120
PgCad1_R4         PAEPRDDVCINGWYPQLTSTSLGTIIIHMEEEIEGDVAIAKLNYDGSGTPEIVQPMVIGS 120
                  ************************************************************

AY19373.1_R1      FNLLSPEIRNENGAWYLYITNRQDYETPTMRRYTFDVRVPDETRAARVSLSIENIDDNDP 180
AY19374.1_R2      FNLLSPEIRNENGAWYLYITNRQDYETPTMRRYTFDVRVPDETRAARVSLSIENIDDNDP 180
AY19375.1_R3      FNLLSPEIRNENGAWYLYITNRQDYETPTMRRYTFDVRVPDETRAARVSLSIENIDDNDP 180
AY198374.1_S      FNLLSPEIRNENGAWYLYITNRQDYETPTMRRYTFDVRVPDETRAARVSLSIENIDDNDP 180
PgCad1_R4         FNLLSPEIRNENGAWYLYITNRQDYETPTMRRYTFDVRVPDETRAARVSLSIENIDDNDP 180
                  ************************************************************

AY19373.1_R1      IVRVLDACQVPELGEPRLTDCVYQVSDEDGRLSIEPMTFRLTSDREDVQIFYVEPAHITG 240
AY19374.1_R2      IVRVLDACQVPELGEPRLTDCVYQVSDEDGRLSIEPMTFRLTSDREDVQIFYVEPAHITG 240
AY19375.1_R3      IVRVLDACQVPELGEPRLTDCVYQVSDEDGRLSIEPMTFRLTSDREDVQIFYVEPAHITG 240
AY198374.1_S      IVRVLDACQVPELGEPRLTDCVYQVSDEDGRLSIEPMTFRLTSDREDVQIFYVEPAHITG 240
PgCad1_R4         IVRVLDACQVPELGEPRLTDCVYQVSDEDGRPSIEPMTFRLTSDREDVQIFYVEPAHITG 240
                  ****************************** *****************************

AY19373.1_R1      DWFNMQITIGILSALNFESNPLHIFQITALDSWPNNHTVTVMVQVQNVEHRPPRWMEIFA 300
AY19374.1_R2      DWFNMQITIGILSALNFESNPLHIFQITALDSWPNNHTVTVMVQVQNVEHRPPRWMEIFA 300
AY19375.1_R3      DWFNMQITIGILSALNFESNPLHIFQITALDSWPNNHTVTVMVQVQNVEHRPPRWMEIFA 300
AY198374.1_S      DWFNMQITIGILSALNFESNPLHIFQITALDSWPNNHTVTVMVQVQNVEHRPPRWMEIFA 300
PgCad1_R4         DWFNMQITIGILSALNFESNPLHIFQITALDSWPNNHTVTVMVQVQNVEHRPPRWMEISA 300
                  ********************************************************** *

AY19373.1_R1      VQQFDEMTEQQFQVRAIDGDTGIGKAIHYTLETDEEEDLFFIETLPGGHDGAIFSTAMID 360
AY19374.1_R2      VQQFDEMTEQQFQVRAIDGDTGIGKAIHYTLETDEEEDLFFIETLPGGHDGAIFSTAMID 360
AY19375.1_R3      VQQFDEMTEQQFQVRAIDGDTGIGKAIHYTLETDEEEDLFFIETLPGGHDGAIFSTAMID 360
AY198374.1_S      VQQFDEMTEQQFQVRAIDGDTGIGKAIHYTLETDEEEDLFFIETLPGGHDGAIFSTAMID 360
PgCad1_R4         VQQFDEMTEQQFQVRAIDGDTGIGKAIHYTLETDEEEDLFLIETLPGGHDGAIFSTAMID 360
                  ****************************************:*******************

AY19373.1_R1      VDRLRRDVFRLSLVAYKYDNVSFATPTPVVIIVNDINNKKPQPLQDEYTISIMEETPLSL 420
AY19374.1_R2      VDRLRRDVFRLSLVAYKYDNVSFATPTPVVIIVNDINNKQPQPLQDEYTISIMEETPLSL 420
AY19375.1_R3      VDRLRRDVFRLSLVAYKYDNVSFATPTPVVIIVNDINNKKPQPLQDEYTISIMEETPLSL 420
AY198374.1_S      VDRLRRDVFRLSLVAYKYDNVSFATPTPVVIIVNDINNKKPQPLQDEYTISIMEETPLSL 420
PgCad1_R4         VDRLRRDVFRLSLVAYKYDNVSFATPTPVVIIVNDINNKKPQPLQDEYTISIMEETPLSL 420
                  ***************************************:********************

AY19373.1_R1      NFAELFGFYDEDLIYAQFLVEIQGENPPGVEQAFYIAPTAGFQNQTFAIGTQDHRMLDYE 480
AY19374.1_R2      NFAELFGFYDEDLIYAQFLVEIQGENPPGVEQAFYIAPTAGFQNQTFAIGTQDHRMLDYE 480
AY19375.1_R3      NFAELFGFYDEDLIYAQFLVEIQGENPPGVEQAFYIAPTAGFQNQTFAIGTQDHRMLDYE 480
AY198374.1_S      NFAELFGFYDEDLIYAQFLVEIQGENPPGVEQAFYIAPTAGFQNQTFAIGTQDHRMLDYE 480
PgCad1_R4         NFAELFGFYDEDLIYAQFLAEIQGENPPGVEQAFYIAPTAGFQNQTFATGTQDHRMLDYE 480
                  *******************.**************************** ***********

AY19373.1_R1      DVPFQNIKLKVIATDRDNTNFTGVAEVNVNLINWNDEEPIFEEDQLVVKFKETVPKDYHV 540
AY19374.1_R2      DVPFQNIKLKVIATDRDNTNFTGVAEVNVNLINWNDEEPIFEEDQLVVKFKETVPKDYHV 540
AY19375.1_R3      DVPFQNIKLKVIATDRDNTNFTGVAEVNVNLINWNDEEPIFEEDQLVVKFKETVPKDYHV 540
AY198374.1_S      DVPFQNIKLKVIATDRDNTNFTGVAEVNVNLINWNDEEPIFEEDQLVVKFKETVPKDYHV 540
PgCad1_R4         DVPFQNIKLKVIATDRDNTNFTGVAEVNVNLINWNDEEPIFEEDQLVVKFKETVPKDYHV 540
                  ************************************************************
```

```
AY19373.1_R1      GRLRAHDRDIGDSVVHSILGNANTFLRIDEETGDIYVAIDDAFDYHRQNEFNIQVRAQDT 600
AY19374.1_R2      GRLRAHDRDIGDSVVHSILGNANTFLRIDEETGDIYVAIDDAFDYHRQNEFNIQVRAQDT 600
AY19375.1_R3      GRLRAHDRDIGDSVVHSILGNANTFLRIDEETGDIYVAIDDAFDYHRQNEFNIQVRAQDT 600
AY198374.1_S      GRLRAHDRDIGDSVVHSILGNANTFLRIDEETGDIYVAIDDAFDYHRQNEFNIQVRAQDT 600
PgCad1_R4         GRLRAHDRDIGDSVVHSILGNANTFLRIDEETGDIYVAIDDAFDYHRQNEFNIQVRAQGT 600
                  **********************************************************.*

AY19373.1_R1      MSEPESRHTATAQLVIELEDVNNTPPTLRLPRVSPSVEENVPEGFEVNREITATDPDTTA 660
AY19374.1_R2      MSEPESRHTATAQLVIELEDVNNTPPTLRLPRVSPSVEENVPEGFEINREITATDPDTTA 660
AY19375.1_R3      MSEPESRHTATAQLVIELEDVNNTPPTLRLPRVSPSVEENVPEGFEINREITATDPDTTA 660
AY198374.1_S      MSEPESRHTATAQLVIELEDVNNTPPTLRLPRVSPSVEENVPEGFEINREITATDPDTTA 660
PgCad1_R4         MSEPESRHTATARLVIELEDVNDTPPTLRLPRVSPSVEENVPEGFETNREITATDPDTTA 660
                  ************:*********:*********************** *************

AY19373.1_R1      YLQFEIDWDTSFATKQGRDTNPIEFHGCVDIETIFPNPADTREAVGRVVAKEIRHNVTID 720
AY19374.1_R2      YLQFEIDWDTSFATKQGRDTNPIEFHGCVDIETIFPNPADTREAVGRVVAKEIRHNVTID 720
AY19375.1_R3      YLQFEIDWDTSFATKQGRDTNPIEFHGCVDIETIFPNPADTREAVGRVVAKEIRHNVTID 720
AY198374.1_S      YLQFEIDWDTSFATKQGRDTNPIEFHGCVDIETIFPNPADTREAVGRVVAKEIRHNVTID 720
PgCad1_R4         YLQFEIDWDTSFATKQGRDTNPIEFHGCVDIETIFPNPADTREAVGRVVAKEIRHNVTID 720
                  ************************************************************

AY19373.1_R1      FEEFEFLYLTVRVRDLHTDDGRDYDESTFTIIIIDMNDNWPIWASGFLNQTFSIRERSST 780
AY19374.1_R2      FEEFEFLYLTVRVRDLHTDDGRDYDESTFTIIIIDMNDNWPIWASGFLNQTFSIRERSST 780
AY19375.1_R3      FEEFEFLYLTVRVRDLHTDDGRDYDESTFTIIIIDMNDNWPIWASGFLNQTFSIRERSST 780
AY198374.1_S      FEEFEFLYLTVRVRDLHTDDGRDYDESTFTIIIIDMNDNWPIWASGFLNQTFSIRERSST 780
PgCad1_R4         FEEFEFLYLTVRVRDLHTDDGRDYDESTFTIIIIDMNDNWPIWASGFLNQTFSIRERSST 780
                  ************************************************************

AY19373.1_R1      GVVIGSVLATDIDGPLYNQVRYTIIPQEDTPEGLVQIHFVTGQITVDENGAIDADIPPRW 840
AY19374.1_R2      GVVIGSVLATDIDGPLYNQVRYTIIM---------------------------------- 806
AY19375.1_R3      GVVIGSVLATDIDGPLYNQVRYTIIPQEDTPEGLVQIHFVTGQITVDENGAIDADIPPRW 840
AY198374.1_S      GVVIGSVLATDIDGPLYNQVRYTIIPQEDTPEGLVQIHFVTGQITVDENGAIDADIPPRW 840
PgCad1_R4         GVVIGSVLATDIDGPLYNQVRYTIIPQEDTPEGLVQIHFVTGQITVDENGAIDADIPPRW 840
                  *************************

AY19373.1_R1      HLNYTVIASDKCSEENEENCPPDPVFWDTLRDNVINIVDINNKVPAADLSRFNETVYIYE 900
AY19374.1_R2      ------------------------------------------------------------
AY19375.1_R3      HLNYTVIASDKCSEENEENCPPDPVFWDTLGDNVINIVDINNKVPAADLSRFNETVYIYE 900
AY198374.1_S      HLNYTVIASDKCSEENEENCPPDPVFWDTLGDNVINIVDINNKVPAADLSRFNETVYIYE 900
PgCad1_R4         HLNYTVIASDKCSEENEENCPPDPVFWDTLGDNVINIVDINNKVPAADLSRFNETVYIYE 900

AY19373.1_R1      NAPDFTNVVKIYSIDEDRDEIYHTVRYQINYAVNQRLRDFFAIDLDSGQVYVENTNNELL 960
AY19374.1_R2      ------------------------------------------------------------
AY19375.1_R3      NAPDFTNVVKIYSIDEDRDEIYHTVRYQINYAVNQRLRDFFAIDLDSGQVYVENTNNELL 960
AY198374.1_S      NAPDFTNVVKIYSIDEDRDEIYHTVRYQINYAVNQRLRDFFAIDLDSGQVYVENTNNELL 960
PgCad1_R4         NAPDFTNVVKIYSIDEDRDEIYHTVRYQINYAVNQRLRDFFA-----GEVYVENTNNELL 955

AY19373.1_R1      DRDRGEDQHRIFINLIDNFYSEGDGNRNVNTTEVLVILLDENDNAPELPTPEELSWSISE 1020
AY19374.1_R2      ------------------------------------------------------------
AY19375.1_R3      DRDRGEDQHRIFINLIDNFYSEGDGNRNVNTTEVLVILLDENDNAPELPTPEELSWSISE 1020
AY198374.1_S      DRDRGEDQHRIFINLIDNFYSEGDGNRNVNTTEVLVILLDENDNAPELPTPEELSWSISE 1020
PgCad1_R4         DRDRGEDQHRIFINLIDNFYREGDGNRNVNTTEVLVILLDENDNAPELPTPEELSWSISE 1015

AY19373.1_R1      NLQEGITLDGESDVIYAPDIDEEDTPNSHVGYAILAMTVTNRDLDTVPRLLNMLSPNNVT 1080
AY19374.1_R2      ------------------------------------------------------------
AY19375.1_R3      NLQEGITLDGESDVIYAPDIDEEDTPNSHVGYAILAMTVTNRDLDTVPRLLNMLSPNNVT 1080
AY198374.1_S      NLQEGITLDGESDVIYAPDIDEEDTPNSHVGYAILAMTVTNRDLDTVPRLLNMLSPNNVT 1080
PgCad1_R4         NLQEGITLDGESDVIYAPDIDEEDTPNSHVGYAILAMTVTNRDLDTVPRLLNMLSPNNVT 1075

AY19373.1_R1      GFLQTAMPLRGYWGTYDISVLAFDHGIPQQISHEVYELEIRPYNYNPP--------RRLR 1132
AY19374.1_R2      ------------------------------------------------------------
AY19375.1_R3      GFLQTAMPLRGYWGTYDIS----------------------------------------- 1099
AY198374.1_S      GFLQTAMPLRGYWGTYDISILAFDHGIPQQISHEVYELEIRPYNYNPPQFVFPESGTILR 1140
PgCad1_R4         GFLQTAMPLRGYWGTYDISILAFDHGIPQQISHEVYELEIRPYNYNPPQFVFPESGTILR 1135
```

```
AY19373.1_R1      LALERAVVNNVLSLVNGDLLDRIQAIDDDGLDAGVVTFDIVGDADASNYFRVNNDGDNFG 1192
AY19374.1_R2      ------------------------------------------------------------
AY19375.1_R3      -VLERAVVNNVLSLVNGDPLDRIQAIDDDGLDAGVVTFDIVGDADASNYFRVNNDGDNFG 1158
AY198374.1_S      LALERAVVNNVLSLVNGDPLDRIQAIDDDGLDAGVVTFDIVGDADASNYFRVNNDGDNFG 1200
PgCad1_R4         LALERAVVNNVLSLVNGDPLDRIQAIDDDGLDAGVVTFDIVGDADASSYFRVNNDGDNFG 1195

AY19373.1_R1      TLLLTQALPEEGKEFEVTIRATDGGTEPRSYSTDSTITVLFVPTLGDPIFQDNTYSVAFF 1252
AY19374.1_R2      ------------------------------------------------------------
AY19375.1_R3      TLLLTQALPEEGKEFEVTIRATDGGTEPRSYSTDSTITVLFVPTLGDPIFQDNTYSVAFF 1218
AY198374.1_S      TLLLTQALPEEGKEFEVTIRATDGGTEPRSYSTDSTITVLFVPTLGDPIFQDNTYSVAFF 1260
PgCad1_R4         TLLLTQALPEEGKEFEVTIRATDGGTEPRSYSTDSTITVLFVPTLGDPIFQDNTYSVAFF 1255

AY19373.1_R1      EKEVGLTERFSLPHAEDPKNKLCTDDCHDIYYRIFGGVDYEPFDLDPVTNVIFLKSELDR 1312
AY19374.1_R2      ------------------------------------------------------------
AY19375.1_R3      EKEVGLTERFSLPHAEDPKNKLCTDDCHDIYYRIFGGVDYEPFDLDPVTNVIFLKSELDR 1278
AY198374.1_S      EKEVGLTERFSLPHAEDPKNKLCTDDCHDIYYRIFGGVDYEPFDLDPVTNVIFLKSELDR 1320
PgCad1_R4         EKEVGLTERFSLPHAEDPKNKLCTDDCHDIYYRIFGGVDYEPFDLDPVTNVIFLKSELDR 1315

AY19373.1_R1      ETTATHVVQVAASNSPTGGGIPLPGSLLTVTVTVREADPRPVFEQRLYTAGISTSDNINR 1372
AY19374.1_R2      ------------------------------------------------------------
AY19375.1_R3      ETTATHVVQVAASNSPTGGGIPLPGSLLTVTVTVREADPRPVFEQRLYTAGISTSDNINR 1338
AY198374.1_S      ETTATHVVQVAASNSPTGGGIPLPGSLLTVTVTVREADPRPVFEQRLYTAGISTSDNINR 1380
PgCad1_R4         ETTATHVVQVAASNSPTGGGIPLPGSLLTVTVTVREADPRPVFEQRLYTAGISTSDNINR 1375

AY19373.1_R1      ELLTVRATHSENAQLTYTIEDGSMVVDSTLEAVKDSAFHLNAQTGVLILRIQPTASMQGM 1432
AY19374.1_R2      ------------------------------------------------------------
AY19375.1_R3      ELLTVRATHSENAQLTYTIEDGSMAVDSTLEAVKDSAFHLNAQTGVLILRIQPTASMQGM 1398
AY198374.1_S      ELLTVRATHSENAQLTYTIEDGSMAVDSTLEAVKDSAFHLNAQTGVLILRIQPTASMQGM 1440
PgCad1_R4         ELLTVRATHSENAQLTYTIEDGSMAVDSTLEAVKDSAFHLNAQTGVLILRIQPTASMQGM 1435

AY19373.1_R1      FEFNVIATDPDEKTDTAEVKVYLISSQNRVSFIFLNDVETVESNRDFIAETFSVGFNMTC 1492
AY19374.1_R2      ------------------------------------------------------------
AY19375.1_R3      FEFNVIATDPDEKTDTAEVKVYLISSQNRVSFIFLNDVETVESNRDFIAETFSVGFNMTC 1458
AY198374.1_S      FEFNVIATDPDEKTDTAEVKVYLISSQNRVSFIFLNDVETVESNRDFIAETFSVGFNMTC 1500
PgCad1_R4         FEFNVIATDPDEKTDTAEVKVYLISSQNRVSFIFLNDVETVESNRDFIAETFSVGFNMTC 1495

AY19373.1_R1      NIDQVLPGTNDAGVIQEAMAEVHAHFIQDNIPVSADSIEELRSDTQLLRSVQGVLNQRLL 1552
AY19374.1_R2      ------------------------------------------------------------
AY19375.1_R3      NIDQVLPGTNDAGVIQEAMAEVHAHFIQDNIPVSADSIEELRSDTQLLRSVHGVLNQRLL 1518
AY198374.1_S      NIDQVLPGTNDAGVIQEAMAEVHAHFIQDNIPVSADSIEELRSDTQLLRSVQGVLNQRLL 1560
PgCad1_R4         NIDQVLPGTNGAGVIQEAMAEVHAHFIQDNIPVSADSIEELRSDTQLLRSVQGVLNQRLL 1555

AY19373.1_R1      VLNDLVTGVSPDLGTAGVQITIYVLAGLSAILAFLCLILLITFIVRTRALNRRLEALSMT 1612
AY19374.1_R2      ------------------------------------------------------------
AY19375.1_R3      VLNDLVTGVSPDLGTAGVQITIYVLAGLSAILAFLCLILLITFIVRTRALNRRLEALSMT 1578
AY198374.1_S      VLNDLVTGVSPDLGTAGVQITIYVLAGLSAILAFLCLILLITFIVRTRALNRRLEALSMT 1620
PgCad1_R4         VLNDLVTGVSPDLGTAGVQITIYVLAGLSAILAFLCLILLITFIVRTRALNRRLEALSMT 1615

AY19373.1_R1      KYGSVDSGLNRVGIAAPGTNKHAIEGSNPIWNEQIKAPDFDAISDTSDESDLIGIEDLPQ 1672
AY19374.1_R2      ------------------------------------------------------------
AY19375.1_R3      KYGSVDSGLNRVGIAAPGTNKHAIEGSNPIWNEQIKAPDFDAISDTSDESDLIGIEDLPQ 1638
AY198374.1_S      KYGSVDSGLNRVGIAAPGTNKHAIEGSNPIWNEQIKAPDFDAISDTSDESDLIGIEDLPQ 1680
PgCad1_R4         KYGSVDSGLNRVGIAAPGTNKHAIEGSNPIWNEQIKAPDFDAISDTSDESDLIGIEDLPQ 1675

AY19373.1_R1      FKSDYFPPEDSESAHAAFSDRTPRGNDAPIAHSSNNFGFNTSPFSAEFTNRRMRP 1727
AY19374.1_R2      -------------------------------------------------------
AY19375.1_R3      FRSDYFPPEDSESAHAAFSDRTPRGNDAPIAHSSNNFGFNTSPFSAEFTNRRMRP 1693
AY198374.1_S      FKSDYFPPEDSESAHAAFSDRTPRGNDAPIAHSSNNFGFNTSPFSAEFTNRRMRP 1735
PgCad1_R4         FKSDYFPPEDSESAHAAFSDRTPRGNDAPIAHSSNNFGFNTSPFSAEFTNRRMRP 1730
```
